# Spatial transcriptomics reveals injury-responsive compartments and coordinated immune–fibrotic signaling in ANCA-associated renal vasculitis

**DOI:** 10.3389/fimmu.2026.1884100

**Published:** 2026-07-31

**Authors:** Yucheng Tang, Chunhua Zhu, Shuang Chen, Huimei Chen, Hai Qian, Aihua Zhang, Enrico Petretto

**Affiliations:** 1 Institute for Big Data and Artificial Intelligence in Medicine, School of Science, China Pharmaceutical University, Nanjing, Jiangsu, China; 2 Department of Nephrology, Children’s Hospital of Nanjing Medical University, Nanjing, Jiangsu, China; 3 Jiangsu Key Laboratory of Pediatrics, Nanjing Medical University, Nanjing, Jiangsu, China; 4 Programme in Cardiovascular and Metabolic Disorders (CVMD), Duke-NUS Medical School, Singapore, Singapore; 5 Duke-NUS AI + Medical Sciences Initiative (DAISI), Duke-NUS Medical School, Singapore, Singapore; 6 Centre for Biomedical Data Science (CBDS), Duke-NUS Medical School, Singapore, Singapore; 7 Centre of Drug Discovery & State Key Laboratory of Natural Medicines, China Pharmaceutical University, Nanjing, Jiangsu, China

**Keywords:** ANCA-associated vasculitis, CXCR4, immune infiltration, kidney fibrosis, lumican, spatial transcriptomics

## Abstract

**Objectives:**

ANCA-associated vasculitis (AAV) presents with rapidly progressive glomerulonephritis, yet the tissue-level molecular landscape in pediatric AAV remains poorly understood. We aimed to characterize spatially resolved renal disease programs across different histopathological stages of pediatric AAV and compared with adult AAV.

**Methods:**

We applied spatial transcriptomics to renal biopsies from pediatric patients with AAV representing distinct histological classes defined by the Berden classification (4 control, 2 focal, and 3 non-focal cases), generating an *in situ* map of localized disease programs. Gene co-expression network and differential expression analyses were used to identify compartment-specific transcriptional programs and cell type markers, which were correlated with pathological indicators. Functional pathway and ligand–receptor interaction analyses were conducted to characterize lesion-associated signaling. Independent adult AAV spatial transcriptomic cohorts and immunofluorescence were used for validation.

**Results:**

Four disease-associated compartments—immune/interstitial fibroblasts (IM/Fib), glomeruli, myofibroblasts, and vascular compartments—showed distinct compartment-specific signatures that correlated with patient-level histopathology. We further identified coordinated immune–fibrotic signaling associated with different histopathological classes. Integrative analysis with an independent adult AAV spatial transcriptomic cohort revealed conserved core injury programs in glomerular and vascular compartments across age groups, layered with distinct age-dependent immunometabolic and stress-response signatures. Within IM/Fib, the CXCR4–CD74 receptor complex co-localized with IgM^+^ cells, and lumican (LUM) co-localized with collagen I/III; both were validated by immunofluorescence and associated with fibrotic injury. These findings were further corroborated using a larger dataset (n=38) of adult AAV patients.

**Discussion:**

These tissue-anchored signatures represent potential disease-associated molecular features with possible diagnostic relevance, and highlight the value of this foundational spatial transcriptomics map for generating testable hypotheses to be further evaluated in larger, stratified, treatment-annotated cohorts.

## 1 Introduction

Anti-neutrophil cytoplasmic antibodies (ANCA)-associated vasculitis (AAV) represents a group of rare systemic autoimmune diseases, characterized by inflammation of the small-to-medium-sized blood vessels (1). Renal involvement occurs in > 75% of patients, predominantly manifesting as rapidly progressive glomerulonephritis (2). In pediatric AAV, renal involvement is more severe; in China, approximately one-third of children progressing to end-stage renal disease and more than half developing chronic kidney disease (3). However, children exhibit a higher capacity for renal function recovery compared to adults, which is potentially attributable to the greater plasticity of the immune system and the potential for earlier therapeutic intervention (4).

A central immunological mechanism of AAV involves the breakdown of immune tolerance to neutrophil granule proteins, primarily proteinase 3 (PR3) or myeloperoxidase (MPO), resulting in the production of ANCA (5). These ANCAs activate neutrophils, promoting vascular injury through the formation of neutrophil extracellular traps, oxidative burst, and degranulation (6). Activated neutrophils also release autoantigens into the extracellular space, where they are captured by dendritic cells and presented to naïve T cells (7). This leads to the expansion of effector T cells, which infiltrate renal tissue and contribute to crescent formation, interstitial fibrosis, and tubular atrophy (8).

Transcriptomic studies in AAV have identified key immune cell populations and provided insights into disease mechanisms. Single-cell analyses have highlighted disease-associated monocyte subsets (FCGR3A^+^ and FCGR1A^+^) (9), SPP1^+^ lipid-associated macrophages that are associated with inflammation and fibrosis (10), and cytotoxic T cells exhibiting pathogenic profiles in renal tissue (11). Activation of the alternative complement pathway has been demonstrated in AAV (12). Collectively, prior studies primarily focused on circulating immune cells, leaving unresolved how immune and stromal programs are organized within kidney tissue and how this spatial organization relates to injury severity and repair in AAV.

Current AAV treatments rely on immunosuppressive therapy, typically cyclophosphamide with high-dose glucocorticoids, which improves survival but increases infection and malignancy risk (13). The use of the anti-CD20 monoclonal antibody, rituximab, yields comparable renal outcomes, with more effective B-cell depletion and better ANCA seroconversion (14). Avacopan is an oral C5a receptor antagonist that effectively blocks the activation of neutrophils and the release of neutrophil extracellular traps, thereby reducing inflammation and tissue damage in AAV (15). It was authorized for adjunct use with cyclophosphamide or rituximab, combined with a glucocorticoid dosage lower than standard protocols. Yet 10–30% of patients fail to achieve remission, remain glucocorticoid dependent, or progress despite therapy (16), and multiple immune-targeted agents are still in development (11, 17, 18). Although contemporary AAV therapies have largely been established through adult clinical trials, their application in pediatric patients is limited by disease rarity and the lack of age-specific evidence, leading to reliance on adult-derived treatment strategies (19, 20). Collaborative initiatives such as the Childhood Arthritis and Rheumatology Research Alliance have developed consensus treatment plans for severe pediatric AAV, while disease activity can be systematically assessed using the Paediatric Vasculitis Activity Score (19, 21). In addition, guidelines from the Pediatric Subspecialty Group of the Chinese Medical Association integrate evidence with pediatric-specific adaptations, providing a standardized framework for the diagnosis, risk stratification, and management of pediatric AAV (22). Since current therapies improve survival yet leave a substantial fraction of patients with persistent activity, glucocorticoid dependence, and progression to chronic kidney disease, there is a critical need for tissue-level biomarkers that quantify severity and resolve immune–fibrotic signaling *in situ*. We therefore hypothesized that spatially resolved transcriptomic signatures within the renal parenchyma would reveal physiologically relevant biomarkers and pathogenic circuits not detectable in blood. To test this hypothesis, we profiled rare, biopsy-limited renal cortex specimens from pediatric patients with AAV using 10x Genomics Visium, defined AAV-associated regions and constituent cell types, and linked regional programs to focal and non-focal histopathological classes as defined by the Berden classification. We identified markers of AAV-associated regions associated with renal disease pathology and prioritized ligand–receptor interactions linked to histopathological class, which reflects differences in glomerular preservation, together revealing potential therapeutic targets in ANCA-associated vasculitis. Finally, to assess the reproducibility and clinical relevance of the identified cellular communication networks, we replicated the key ligand–receptor interactions observed in the pediatric immune/interstitial fibroblast compartment in a larger independent adult AAV cohort.

## 2 Materials and methods

### 2.1 Ethical compliance

This study complied with all relevant ethical regulations. The use of human kidney biopsy specimens was approved by the Research Ethics Committee of the Children’s Hospital of Nanjing Medical University. All experiments involving human samples were conducted in accordance with applicable guidelines and regulations. Written informed consent was obtained from all participants or their legal guardians, and participation was voluntary.

### 2.2 Human kidney cortex biopsy samples in spatial technologies and immunostaining

This study involved renal cortex biopsies from patients with AAV, approved by the Independent Ethics Committee of Children’s Hospital of Nanjing Medical University (Approval #202008089-1). Kidney samples from patients with AAV and controls were analyzed using spatial transcriptomics and immunostaining (clinical details are provided in **Supplementary Table S1**). Histologically normal control biopsies were obtained from patients with minimal glomerular abnormalities, showing no abnormalities on light microscopy and immunofluorescence (**Supplementary Table S1**; **Supplementary Figure S1**). On electron microscopy, focal (approximately 30–40%) podocyte foot process effacement was observed in all control patients, with no electron-dense deposits identified in any glomerular compartments (**Supplementary Figure S1**). According to Berden’s criteria (**Supplementary Table S1**), AAV patients were categorized into two histopathological classes based on the extent of glomerular involvement, including focal class (>50% normal glomeruli) and non-focal classes (crescentic and sclerotic) (23). Cryopreserved kidney sections were utilized for spatial transcriptomic analysis, whereas formalin-fixed paraffin-embedded (FFPE) sections were prepared for immunofluorescence (IF) staining. Due to limited availability, only two focal AAV cases were included for spatial transcriptomic analysis; an additional patient with the same focal glomerulonephritis subtype, classified according to Berden’s criteria, was included for IF staining (**Supplementary Table S1**). All procedures were conducted in accordance with BRISQ guidelines.

### 2.3 Histopathological analysis of renal tissues

Renal tissue samples were fixed in 4% paraformaldehyde at room temperature for 48 hours and processed for histological examination. Serial sections (2 μm) were prepared for Periodic acid–Schiff (PAS) and Masson’s trichrome staining following established protocols (24). Samples were evaluated for glomerular and tubular basement membrane abnormalities and fibrotic changes, and classified according to the Berden system into focal and non-focal classes in ANCA-associated glomerulonephritis (details are provided in **Supplementary Table S1**).

### 2.4 Construction and sequencing of spatial gene expression libraries

Spatial transcriptomic analysis was performed using the Visium HD Spatial Gene Expression platform (10x Genomics). Kidney biopsy samples were processed according to the Visium Spatial Protocols for tissue preparation and optimization (10x Genomics, CG000240, CG000238) and the Gene Expression Reagent Kit (10x Genomics, CG000239). Three Visium slides were sequenced, comprising control (*n* = 4, two sections each), focal (*n* = 2, three and four sections, respectively), and non-focal (*n* = 3, two sections each). Optimal cutting temperature -embedded sections (10 µm) were methanol-fixed, hematoxylin and eosin (H&E)-stained, and imaged using a Leica DMi8 microscope (10x magnification) following manufacturer protocols (10x Genomics, CG000241). After 12 min permeabilization, mRNA was captured in fiducial capture areas of Visium slides. cDNA libraries were generated via second-strand synthesis, and sequenced on a Novaseq X Plus system (Illumina).

### 2.5 Spatial transcriptomic data processing, filtering, and integration

Spatial transcriptomic data were processed using Space Ranger (10x Genomics, v2.0.0) with the GRCh38 reference genome (refdata-gex-GRCh38-2020-A) to generate raw unique molecular identifier (UMI) count matrices, mapped to 55 μm barcoded spatial spots (**Supplementary Table S12**). Gene-spot matrices underwent individual quality assessment and preprocessing in R Studio (v4.3.2) with STUtility (v1.0.0) and Seurat (v5.0.1) (25, 26). For each group (control, focal and non-focal), raw counts, histological images, spatial coordinates, and scaling factors were imported into STUtility. Metadata (group, patient identifiers, and section numbers) were annotated using the “ManualAnnotation” function, and low-quality spots were removed using “subsetSTData” (**Supplementary Table 12**). Metadata were transferred to Seurat objects, and genes detected in <10% of spots were excluded. Mitochondrial content was calculated but not used for filtering due to limited biopsy size and potential mitochondrial alterations in AAV lesions. For each Seurat object, gene-spot matrix was normalized using the “NormalizeData” function, and 2,000 highly variable features were identified using “FindVariableFeatures”. Integration of control, focal, and non-focal groups was performed by identifying integration anchors with canonical correlation analysis via “SelectIntegrationFeatures” and “FindIntegrationAnchors”, followed by “IntegrateData”. The integrated dataset was processed using Seurat’s standard workflow, including scaling (“ScaleData”) and principal component analysis (“RunPCA”). The first nine principal components were selected based on variance inflection points identified by “ElbowPlot” and used for downstream analyses. UMAP embedding was generated using “RunUMAP”, and neighborhood graphs were constructed using “FindNeighbors”. Finally, we applied cluster detection at a resolution of 0.4 by the “FindClusters” function, which identified ten distinct transcriptional clusters within our dataset. The “DimPlot” function was used to display UMAP plots, utilizing the “umap” reduction method and organizing the data by either “cell types” or “groups”, or splitting them as required.

### 2.6 Cell type annotation

Spot-level cell types were assigned by identifying differentially expressed genes (DEGs) for each cluster versus all others using Seurat’s “FindAllMarkers” function (Model-based Analysis of Single-cell Transcriptomics [MAST] method) with a non-parametric Wilcoxon rank sum test: log2 fold change (FC) ≥ 0.25, and adjusted *P*-value ≤ 0.05 (27). Clusters were annotated based on marker genes, cross-referenced with literature markers (**Supplementary Table S2**) and the Kidney Tissue Atlas (28). Ten cell types were identified: proximal tubule (PT: *MIOX*, *ALDOB*), thick ascending limb (TAL: *SLC12A1*, *CLDN10*), distal convoluted tubule (DCT: *SLC12A3*), connecting tubule/principal cell (CT/PC: *AQP2*, *AQP3*), connecting tubule/intercalated cell (CT/IC: *FOXI1*, *ATP6V1B1*), glomerulus (Glo: *NPHS2*, *PTPRO*), immune cell types and interstitial fibroblasts (IM/Fib: *CD74*, *IGHG1*), vascular endothelial cells/smooth muscle cells (vEC/VSMC: *MYH11*, *ACTA2*), myofibroblasts (Myo: *COL8A1*), and mixed tubules. Cellular composition was calculated as the proportion of spots per cell type relative to total spots for inter-group comparison, and per patient relative to total spots for patient-level analysis.

### 2.7 Validation of cell type annotations

To validate the robustness of our marker-based cell-type annotations, we utilized the Giotto package (v3.3.0) to perform hypergeometric enrichment analysis at each spatial spot, using two independent single-cell databases (KCA and DISCO; **Supplementary Table S9**) as references. Furthermore, we employed Robust Cell Type Decomposition (RCTD) deconvolution from the spacexr package (v2.0.0) on the spatial transcriptomic datasets using the KCA dataset to provide an additional layer of cross-verification. Detailed methodological information is provided in the **Supplementary Methods**.

### 2.8 Gene co-expression network analysis

High-dimensional weighted gene co-expression network analysis (hdWGCNA) was performed to identify gene modules associated with AAV-associated cell types and glomerular involvement across focal and non-focal histopathological classes. Analyses were conducted using the hdWGCNA package (v0.3.3) with a soft-thresholding power of 9 (29) on the non-focal AAV group (**Supplementary Figure S7A**). The soft power was selected based on maximization of scale-free topology fit and minimization of mean connectivity, thereby identifying clusters with the strongest intra-group and weakest inter-group connections. Module eigengenes (MEs) represent the first principal component of the gene expression profiles within a co-expression module, serving as a summary of the module’s overall expression pattern. MEs were correlated with cell type annotations using Pearson (point-biserial) correlation, and *P*-values were adjusted by the Benjamini-Hochberg method. Module scores were computed in Seurat using the top 100 co-expressed genes ranked by kME, a measure of gene–module membership. Based on cell type annotation, four modules (M2, M3, M8, and M11) were enriched in AAV-associated cell types and associated with the extent of glomerular involvement. Topological overlap matrices were calculated using “GetTOM” in hdWGCNA. Hub gene networks were visualized in Cytoscape (v3.9.1), and ligand–receptor genes within each module were annotated using the FANTOM5 ligand–receptor database (30).

### 2.9 Differential expression analysis

Differential expression analysis was performed in Seurat using “FindMarkers” with the MAST framework (27) to compare focal vs. control, non-focal vs. control, and non-focal vs. focal groups across cell types. MAST is a generalized linear model framework that models both detection rate and expression level in transcriptomic data and supports covariate-adjusted differential expression analysis. DEGs were defined as log2FC ≥ 0.25, and adjusted *P*-value ≤ 0.05 (Wilcoxon rank sum test).

### 2.10 Identification of AAV-associated regions

AAV-associated regions were defined by two criteria: (1) cell type proportions differed significantly (*P*-value ≤ 0.05) between focal vs. control groups or non-focal vs. control groups; (2) AAV marker genes (**Supplementary Table S4**) were expressed in disease but not controls, with scaled average expression > 0 in both focal and non-focal groups (**Supplementary Table S5**; **Supplementary Figure S4B**).

**Figure 1:**
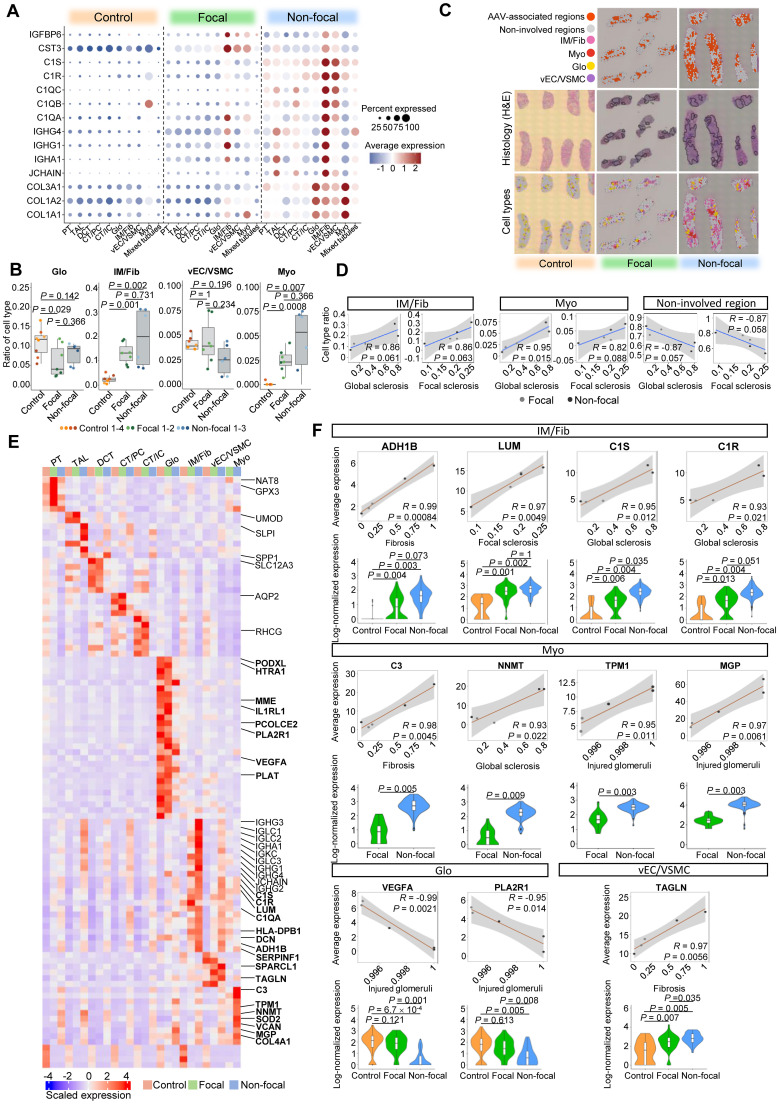
Identify and characterize AAV-associated regions associated with histological stratification based on focal and non-focal glomerular involvement. **(A)** Dot plot of injury marker expression across control, focal, and non-focal AAV groups. **(B)** Boxplots showing proportions of AAV-associated cell types in patient samples across control, focal, and non-focal groups; colors indicate individual patients. *P*-values were calculated using Wilcoxon rank-sum test at the sample level. **(C)** Highlighted AAV-associated regions, corresponding histology, and spot-enriched cellular compositions in AAV samples. Control samples are shown for comparison. **(D)** Scatter plot illustrates the correlation between the pathological indicators of kidney sections from AAV patients (x-axis) and the ratios of representative regions (y-axis) in five AAV patient samples. Non-involved regions encompass spot-enriched cellular compositions lacking AAV-associated regions. **(E)** Heatmap showing the expression of cell type markers across control, focal, and non-focal AAV groups. Representative markers for each cell type are indicated, with those correlated to pathological indicators from AAV-associated regions highlighted in bold (Spearman’s correlation: *P*-value ≤ 0.05). **(F)** Scatter plots showing the relationships between kidney pathology indicators in AAV patients (x-axis) and expression levels of key cell type markers in AAV-associated regions (y-axis). Violin plots compare the expression levels of these markers across control, focal, and non-focal groups within each AAV-associated region. *P*-values were determined using Wilcoxon rank-sum test at the sample level.

Although vEC/VSMC contained relatively few spots and showed no significant changes in proportion, they were included as AAV-associated region because they represent vascular cell populations directly involved in vasculitis and exhibit AAV-associated injury-related gene expression patterns (**Figure 1A**; **Supplementary Figure S5B, C**). Besides, mixed tubules were excluded because the spatial transcriptomic resolution/quality was insufficient to distinguish tubular subtypes. In summary, we defined IM/Fib, Myo, Glo, and vEC/VSMC as AAV-associated regions, which meet the criteria above.

### 2.11 Association between cell type and pathological indicators

Associations between cell type proportions from integrated spatial transcriptomic dataset and pathological indicators from patient biopsies was assessed using Spearman correlation using the ‘cor’ function from the stats package (v4.3.2), with significance determined via *P*-values within 95% confidence intervals from the ‘cor.mtest’ function in the corrplot package (v0.95). Pathological indicators were derived from quantitative biopsy metrics obtained from diagnostic biopsies (**Supplementary Table S1**). Because controls lacked glomerular injury and fibrosis measures, analyses were restricted to AAV samples. Results were visualized as a clustered heatmap by the ‘pheatmap’ function, with rows as pathological indicators, columns as cell types, and color intensity indicating the Spearman correlation coefficient (**Supplementary Figure S4E**). Statistical significances were annotated with asterisks (* *P*-value ≤ 0.1, ** *P*-value ≤ 0.05) and hierarchical clustering to identify cell types potentially linked to clinical features.

### 2.12 Correlation between cell type markers/DEGs and pathological indicators

Correlations between cell-type-specific marker genes/DEGs and pathological indicators were evaluated by ‘lm’ function from R stats package. For each gene-pathology indicator pair, regression models were fitted, with *R*² and *P*-values. Correlations meeting dual thresholds (*P*-value < 0.05 and *R*² > 0.2) were considered biologically meaningful.

### 2.13 Functional enrichment analysis

To identify biological pathways associated with each cell type, over-representation analysis was performed on commonly upregulated DEGs using the clusterProfiler package (v4.8.3) (31). Enrichment was conducted against Gene Ontology Biological Process terms (32), and redundant gene ontology terms were removed using the ‘simplify’ function (similarity cutoff = 0.6). Pathways with *q*-value ≤ 0.05 were considered significant. The same approach was applied to WGCNA modules and ligand–receptor gene sets in AAV-associated regions. Gene set enrichment analysis (GSEA) was performed to assess functional enrichment of DEGs in AAV-associated regions (31, 33), with genes ranked by average log2 fold change from each AAV-associated cell type. Analyses were conducted using the “gsePathway” function in the ReactomePA package (v1.44.0) (34). Normalized enrichment scores (NES) were derived based on gene set overlap with Reactome canonical pathways. Multiple testing correction was applied using the Benjamini-Hochberg method, with significance defined as false discovery rate (FDR) ≤ 20% and minimum gene set size (minGSSize) of 10. Results were visualized using the circlize package (v0.4.16).

### 2.14 Validation using external adult AAV ST datasets

To determine whether the key molecular features and injury-responsive programs identified in pediatric AAV are also observed in adults, we assessed their generalizability using an independent adult kidney spatial transcriptomic (Visium) cohort from Germany comprising 19 patients with AAV (**Supplementary Table S9**) (11). Using the fgsea package (v1.26.0), we evaluated the enrichment of reference-derived cell-type signatures within our spatial compartments. Key markers identified in the pediatric AAV dataset were further assessed for consistency. In addition, cross-cohort transcriptomic comparison between pediatric and adult samples—including data integration, differential gene expression analysis, and functional pathway enrichment—to assess the robustness of the findings. Detailed methodological information and reference descriptions are provided in the **Supplementary Methods**.

**Figure 2:**
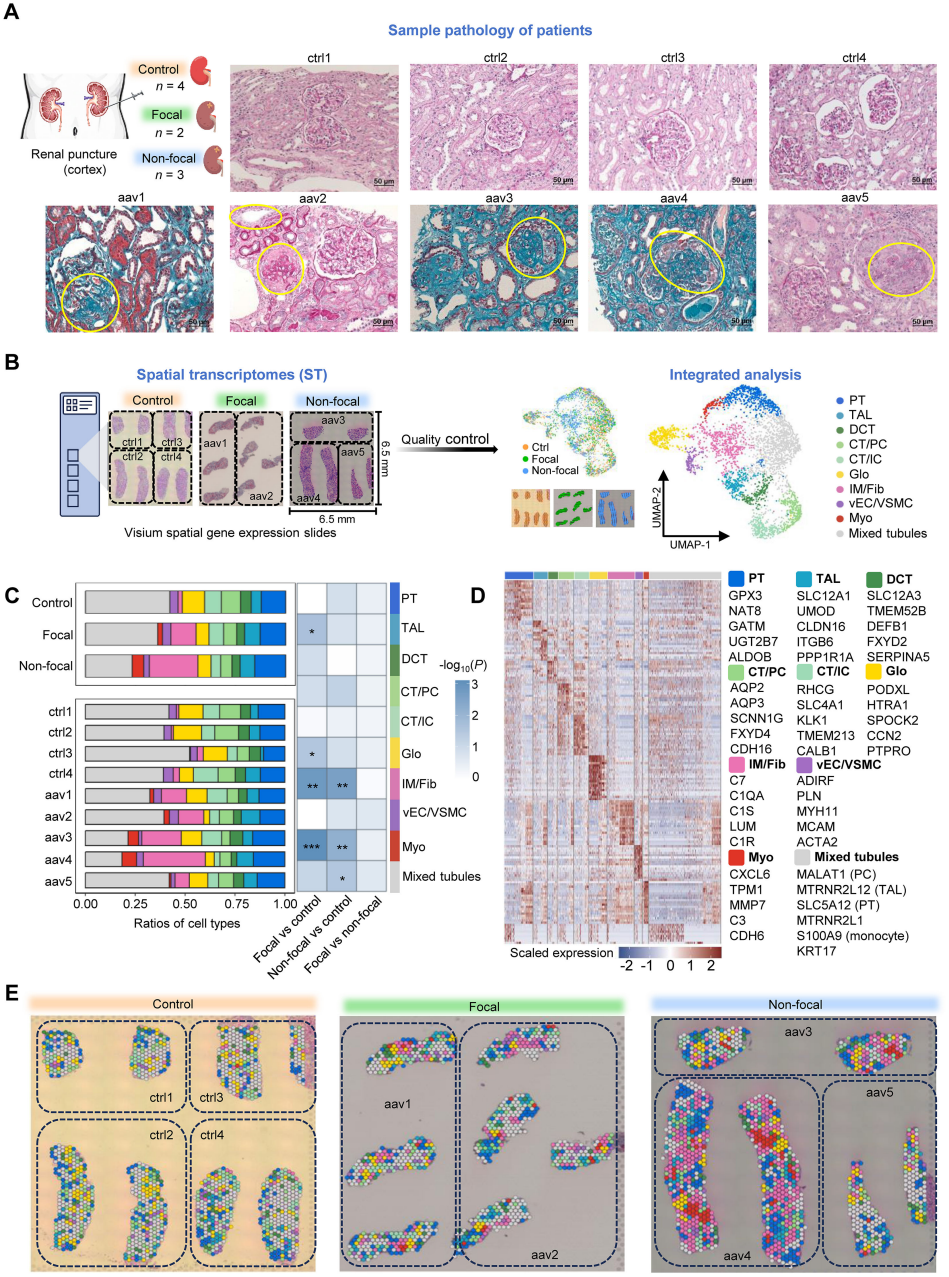
Study design and clinical characterization of control (ctrl), focal and non-focal ANCA-associated vasculitis (AAV) datasets. **(A)** Histological staining of renal cortex tissues from control, focal AAV, and non-focal AAV groups. Images were obtained from 4 control, 2 focal AAV and 3 non-focal AAV patients. Control samples are labeled ‘ctrl’. The aav1 case (focal) shows segmental glomerular sclerosis (light microscopy, Masson trichrome). The aav2 case (focal) exhibits global glomerulosclerosis with tubular atrophy (light microscopy, PAS). The aav3–5 cases (non-focal) display crescentic glomerular lesions (light microscopy; aav3 & aav4, Masson trichrome; aav5, PAS). Yellow circles highlight pathological regions. Scale bars, 50 μm. **(B)** Schematic workflow showing the study design and spatial transcriptome analysis. Tissue sections were overlaid on Visium slides, sequenced, and subjected to quality control and integrated downstream analysis. UMAP visualization shows unsupervised clustering with annotated cell types: PT, proximal tubule; TAL, thick ascending limb; DCT, distal convoluted tubule; CT/PC, connecting tubule/principal cell; CT/IC, connecting tubule/intercalated cell; Glo, glomerulus; IM/Fib, immune cell and fibroblast; vEC/VSMC, vascular endothelial cell/vascular smooth muscle cell; Myo, myofibroblast. **(C)** Cell type proportions across groups and patients (left). Differences among groups were assessed by Wilcoxon rank-sum test, with –log10(*P*-value) values shown in the heatmap. *P-value ≤ 0.05, **P-value ≤ 0.01, ***P-value ≤ 0.001 (right). **(D)** Heatmap showing the top 20 differentially expressed genes (DEGs) for each cell type in the integrated datasets, with five representative markers are highlighted per cell type. **(E)** H&E images overlaid with annotated cell types in control, focal and non-focal AAV datasets.

To validate whether the key ligand–receptor pairs identified in the IM/Fib region of pediatric AAV were reproducible in adult cohorts, we assessed their interaction scores in an adult kidney spatial transcriptomic (Xenium) cohort from Germany comprising 6 controls and 32 AAV patients (**Supplementary Table S9**) (35). According to the original study, periglomerular niches (Periglo) were characterized by an increased infiltration of innate and adaptive immune cells and were associated with crescent formation (**Supplementary Figure S11A**). This specific niche corresponds to the IM/Fib compartment in our dataset, which is predominantly located around the glomeruli (**Figure 2E**). Hence, ligand–receptor interaction score matrices for the periglomerular region were generated and analyzed following the NICHES workflow below. Interactions (CD74–CXCR4, LUM–ITGB1, and COL3A1–ITGB1) detected in at least three AAV patients were retained. To prioritize cell–cell interactions by strength and robustness, we defined a composite score integrating interaction magnitude and patient-level support, calculated as: mean_score × prop_positive × log1p(n_positive), where mean_score is the average interaction score across samples, prop_positive is the proportion of patients with detectable signals, and n_positive is the number of supporting patients. The top 10 ranked interactions were visualized in a heatmap. Gene expression levels were compared between control and AAV patients using the non-parametric Wilcoxon rank-sum test. The absence of COL1A2 in the Xenium panel restricted fibroblast signature validation to COL3A1.

### 2.15 Cell-cell communication analysis using NICHES

Ligand-receptor (L-R) cell-cell communication analysis was performed using the NICHES package (v1.0.0) (36). NICHES analyzes the gene expression matrix with spot metadata from spatial transcriptomic datasets as input and generates interaction matrices as output using the “RunNICHES” function. Interaction matrices were generated based on ligand–receptor pairs curated from the OmniPath database (37), with interaction strength defined as the product of ligand expression in sender cells and receptor expression in receiver cells. Differential L–R interactions between AAV and control groups were assessed using Seurat “FindMarkers” with a Wilcoxon rank-sum test, applying thresholds of log2FC ≥ 0.25 and adjusted *P*-value ≤ 0.05. Interaction networks were constructed using the InterCellar package (v2.6.0) (38), where nodes represent cell types and edges denote autocrine or paracrine interaction frequencies.

### 2.16 Immunofluorescence staining

IF staining was conducted on 3-μm paraffin-embedded kidney sections from control, focal and non-focal AAV groups (*n* = 3 per group). For triple-labeling, sections were processed using a TSA-based four-color kit (Abclonal, Research Resource Identifiers [RRID]: RK05903). IF staining was initiated by deparaffinization, with slides baked at 65 °C for 30 min, followed by sequential immersion in xylene (37 °C), absolute ethanol, and graded ethanol (95%, 85%, 70%) for rehydration, and rinsing in PBS. Antigen retrieval was performed in Tris-EDTA buffer (pH 9.0, Beyotime, RRID: P0084) using an antigen retrieval cooker (Aptum, 2100 Retriever) for 20 min, followed by cooling to room temperature and PBS washing. Endogenous peroxidase activity was quenched with 3% H_2_O_2_ (ORIGENE, RRID: PV-9000) for 20 min at RT, followed by PBS rinsing and blocking with 5% BSA (Beyotime, RRID: P0102) for 1 h at RT.

Primary antibodies were applied sequentially over three days: day 1, anti-CXCR4 (Abcam, RRID: ab124824, 1:100); day 2, anti-CD74 (Invitrogen, RRID: 14-0747-82, 1:100); and day 3, anti-IgM (ORIGENE, RRID: 24052901, 1:30, direct green fluorescence without secondary antibody). Primary antibodies (excluding anti-IgM) were incubated overnight at 4 °C, followed by washing and incubation with HRP-conjugated secondary antibody (Abclonal, RRID: RK05903-5) for 50 min at RT. Signal detection was performed using TSA reagents, with TYR-570 for anti-CD74 (red; Abclonal, RRID: RK05903-2) and TYR-690 for anti-CXCR4 (violet; Abclonal, RRID: RK05903-3), followed by amplification using TSA+enhancer (1:200; Abclonal, RRID: RK05903-4). After each staining round, antibodies were stripped using EDTA buffer at 95 °C for 40 min, and the cycle (peroxidase blocking to TSA) was repeated for subsequent targets. Sections were mounted using antifade mounting medium with DAPI (Vector Laboratories, RRID: H-1200). Slide images were acquired using a Panoramic MIDI slide scanner (3DHISTECH, RRID: PMIDI23C3001) fitted with a Grasshopper3 USB 3.0 camera (FLIR, RRID: GS3-U3-51S5M-C) and a Zeiss 20X Plan-Aprochromat objective. Images were captured at 8-bit depth with and exposure times ranging from 8–60 ms. Post-acquisition processing was performed using CaseViewer (3DHISTECH). Fluorescence signals for each antibody were imaged under identical conditions across groups (200× magnification).

Double-labeling IF staining was performed using a TSA-based three-color kit (Abclonal, RRID: RK05902) following the protocol described above. Primary antibodies were applied sequentially over two days: day 1, anti-lumican (Proteintech, RRID: 10677-1-AP, 1:400) or anti-CXCR4; day 2, anti-collagen I and III (Bioss, RRID: BS-10423R and RRID: BS-0549, 1:400) or anti-CD74. Signal detection was performed using TYR-520 for CXCR4 and lumican antibodies (green; Abclonal, RRID: RK05903-1) and TYR-570 for CD74, collagen I, and collagen III antibodies (red; Abclonal, RRID: RK05903-2). Fluorescence images were acquired using an X-cite Series 120 Q unit (Excelitas, USA) attached to a light microscope Imager A2 (Zeiss, Germany) with an Axiocam 503 camera (Zeiss, Germany). Five random fields per section (200× magnification) were imaged under standardized optical and exposure conditions (150–200 ms). Quantitative analysis was conducted using ImageJ (v1.54k, National Institutes of Health, USA) (39).

### 2.17 Statistical analysis

Statistical analyses were performed using R Studio (v4.3.2) and GraphPad Prism (v10.4.1; GraphPad Software, LLC, www.graphpad.com).

Given the limited number of patients in the focal group (*n* = 2), the biopsy was used as the unit of statistical analysis (**Supplementary Table S11**). Multiple biopsy samples were obtained from each patient. Statistical comparisons were performed using the non-parametric Wilcoxon rank-sum tests. This approach does not assume normality of the underlying data distribution and is less sensitive to outliers, making it well suited for robust statistical inference in small-sample settings. *P*-value ≤ 0.05 was considered statistically significant. *P*-values were adjusted for multiple testing using the Benjamini–Hochberg method where applicable.

### 2.18 Packages for spatial transcriptomic figures

Top DEGs for each cell type were visualized as heatmaps using Seurat “DoHeatmap” on integrated datasets. Additional heatmaps were generated using the pheatmap (v1.0.12) with optimized parameters. Spatial distributions of cell types or AAV-associated regions in control, focal, and non-focal groups were visualized using Seurat “SpatialDimPlot”, “SpatialFeaturePlot” was used to display scaled expression and spatial patterns of key markers in AAV-associated regions. Violin plots were generated using Seurat “VlnPlot”, with the interquartile range (25th–75th percentile) and median (50th percentile) indicated. Dot plots were generated using Seurat “DotPlot” to display gene expression across cell types. Other figures were generated using ggplot2 (v3.5.1) with optimized parameters.

## 3 Results

### 3.1 Spatial transcriptomics of kidney biopsies in ANCA-associated vasculitis

We categorized pediatric AAV patients into focal (*n* = 2) and non-focal (*n* = 3) groups and analyzed kidney cortex biopsies alongside those of controls (*n* = 4) (**Supplementary Table S1**). The histologically normal controls, obtained from patients with minimal glomerular abnormalities, showed no evidence of glomerulosclerosis, tubular atrophy, or immune deposits on light microscopy, immunofluorescence, or electron microscopy (**Figure 2A**; **Supplementary Figure 1**; **Supplementary Table S1**). PAS staining delineated glomerular basement membranes and mesangial expansion, allowing the identification of segmental sclerosis and glomerular crescents, while Masson’s trichrome staining highlighted glomerular fibrosis. In focal AAV biopsies, segmental sclerosis and tubular atrophy were detected, whereas non-focal AAV consistently exhibited cellular glomerular and extensive fibrotic deposition, reflecting greater glomerular involvement accompanied by fibrotic tissue remodeling (**Figure 2A**). None of the patients had received treatment at the time of biopsy collection.

We generated spatial transcriptomics profiles from kidney biopsies of pediatric control and AAV patients using the Visium platform, analyzing 2–4 tissue sections per group (**Figure 2B**). After standardizing quality control, we obtained 3,850 high-quality spots (1,413 in controls; 890 in focal AAV; 1,277 in non-focal AAV). Non-focal AAV samples exhibited increased transcript and gene counts per spot, accompanied by reduced mitochondrial content (**Supplementary Figure S2A**).

Using unsupervised clustering and marker gene expression, we identified 10 distinct cell types from the integrated dataset (**Supplementary Figures S2E**, **3**; **Supplementary Table S2**), which were visualized in a UMAP plot and spatially mapped across kidney sections (**Figures 2B, E**). These included proximal tubule (PT), thick ascending limb (TAL), distal convoluted tubule (DCT), connecting tubule/principal cells (CT/PC), connecting tubule/intercalated cells (CT/IC), glomerulus (Glo), immune cells/interstitial fibroblasts (IM/Fib), vascular endothelial/smooth muscle cells (vEC/VSMC), myofibroblasts (Myo), and mixed tubules.

Our cell type annotations were validated using scRNA-seq references from the Deeply Integrated human Single-Cell Omics data (DISCO) and Kidney Cell Atlas (KCA), showing strong agreement with established marker gene expression (**Supplementary Table S9**; **Supplementary Figure S2C**; see **Supplementary Methods**). As an orthogonal validation, we applied RCTD-based spatial deconvolution using the KCA scRNA-seq reference and evaluated the agreement between RCTD-inferred cell type assignments and annotated cell types (**Supplementary Figures S4A-C**, see **Supplementary Methods**). Concordance was assessed using the Jaccard index based on spot-level set overlap, which ranged from 0.1 to 0.72 across cell types (**Supplementary Figure S4A**). While certain major cell populations showed relatively higher agreement, overall agreement varied across cell types. We further provide the corresponding set sizes and intersection counts in **Supplementary Table S13**.

Cell type distributions were consistent across patients, and, as expected, controls showed fewer IM/Fib cells and almost no Myo cells (**Supplementary Figure S2B**). The IM/Fib and Myo clusters showed the most pronounced changes among cell types in both focal and non-focal AAV patients compared to controls (**Figures 2C, E**), with progressive expansion accompanying greater glomerular involvement and associated pathological features such as immune infiltration and fibrosis. The IM/Fib cluster was most abundant in the non-focal AAV group. In contrast, glomerular (Glo) spots were reduced in AAV samples, especially in the focal group, suggesting a potential link to early glomerular sclerosis. While TAL, CT/PC, and mixed tubule populations showed some variability across conditions, their changes were less prominent. Notably, the mixed tubule cluster co-expressed markers from multiple cell types (e.g., *MALAT1* for PC, *SLC5A12* for PT, *S100A9* for monocytes) (**Figure 2D**), had the lowest transcript and gene counts, and exhibited high mitochondrial content (**Supplementary Figure S2D**), were flagged as low-quality by pre-specified QC thresholds (genes/UMIs/% mito) and excluded *a priori* from downstream analyses.

We identified 2,865 upregulated DEGs across the 10 annotated kidney cell types (**Supplementary Table S3**). The top 20 DEGs showed distinct, cell-type-specific expression patterns (**Figure 2D**), with strong concordance between our spatial transcriptomic data and the scRNA-seq profiles from the Kidney Precision Medicine Project (KPMP) atlas (**Supplementary Table S3**) (28). The IM/Fib and Myo clusters exhibited unique molecular signatures, including enrichment in complement pathway components (*C7*, *C1QA*, *C1S*, *C1R*, *C3*) and extracellular matrix organization (*LUM*, *MMP7*) (**Supplementary Figure S2F**). Myo cells also showed upregulation of genes related to immune response (*CXCR6*), wound healing (*TPM1*), and cell junction assembly (*CDH6*).

This spatial transcriptomic profiling of pediatric AAV kidney biopsies revealed expansion of immune-fibroblast and myofibroblast populations, as well as reduced glomerular signatures and distinct cell-type-specific gene expression programs enriched for complement activation and fibrosis-related pathways.

### 3.2 Delineating pathogenic AAV-associated regions during disease progression

To characterize AAV-associated cell types *in situ*, we defined “disease-associated” regions integrating changes in cell type proportions (**Figure 2C**) with the expression of canonical AAV-related pathological marker genes (**Figure 1A**; **Supplementary Table S4**). The AAV-associated regions highlighted four key cell types – IM/Fib, Myo, Glo, and vEC/VSMC – that were consistently detected across AAV patients, mirroring the distribution of injury markers (**Figures 1A, B**; **Supplementary Figure S5B**). These cell populations were spatially mapped across control, focal, and non-focal AAV groups (**Figure 1C**), showing proximity within tissue sections and adjacent positioning in UMAP space (**Supplementary Figure S5A**), which suggests shared transcriptional features. Consistent with the overall cell-type distribution shown in **Figure 2C**, quantitative analysis confirmed significant alterations in cell-type compositions in both focal and non-focal AAV compared with controls. IM/Fib and Myo populations were increased in both AAV subgroups, with higher mean proportions in the non-focal group, although differences between focal and non-focal AAV were not statistically significant. Glo proportions were reduced relative to controls, without significant differences between the two AAV subgroups. Although vEC/VSMC cells were relatively sparse, they expressed immune-related genes (*C1S*, *C1R*, *C1QA*–*C1QC*, *IGHA1*, *JCHAIN*), injury markers (*IGFBP6*, *CST3*), and profibrotic genes (*COL1A2*, *COL3A1*), overlapping transcriptionally with both immune-fibrotic and glomerular compartments (**Figure 1A**). These four cell types, most strongly associated with AAV progression, were selected for focused downstream analyses.

To assess whether AAV-associated cell types are linked to disease pathology, we correlated cell type proportions from spatial transcriptomic dataset with histopathological indicators from diagnostic kidney biopsies. Among all cell types, IM/Fib and Myo showed the strongest positive associations with key pathological features, including focal/global glomerulosclerosis, glomerular injury, and interstitial fibrosis (**Supplementary Figure S5C**). IM/Fib proportions correlated strongly with both focal and global sclerosis, while Myo showed the highest correlation with global sclerosis (*R* = 0.95, *P* = 0.015) (**Figure 1D**). In contrast, non-involved regions exhibited negative correlations with these pathological measures. While PT cells also showed positive associations with injury, they were not prioritized due to minimal expression of AAV-relevant markers (**Figure 1A**). Glo and vEC/VSMC populations displayed distinct correlation patterns: Glo was positively associated with crescentic glomeruli, whereas vEC/VSMC showed inverse correlations (**Supplementary Figure S5C**). Together, these findings highlight dynamic shifts in IM/Fib and Myo populations, which may reflect their association with glomerular injury and tubulointerstitial fibrosis in AAV.

### 3.3 Markers from AAV-associated regions correlate with pathological indicators

We identified differentially expressed cell type markers across control, focal, and non-focal AAV groups (**Figure 1E**) and highlighted marker genes in AAV-associated cell types (Glo, IM/Fib, Myo, and vEC/VSMC), which showed significant correlations with pathological indicators (*R*² > 0.7, *P* < 0.05) (**Figure 1F**; **Supplementary Figure S6**; **Supplementary Table S6**).

Tubular (e.g., *UMOD*, *SLC12A3*, *AQP2* and *RHCG*) and glomerular (e.g., *PODXL*, *HTRA1*) markers were markedly reduced in non-focal AAV group, reflecting extensive loss of renal architecture (**Figure 1E**). The upregulation of *NAT8*, a key detoxification enzyme, and *GPX3*, a major antioxidant, in focal AAV group suggests an active protective and compensatory responses of surviving proximal tubular cells to moderate injury (40, 41), while their diminished expression in non-focal AAV might indicate failure of these renal protective pathways (42). In non-focal AAV tubular cells, the upregulation of *SLPI* reflects a protective, anti-protease function against tissue injury mediated by neutrophil enzymes (43). Conversely, increased *SPP1* expression suggests pro-inflammatory and pro-fibrotic signaling activation contributing to chronic tubulointerstitial damage and scarring (44).

Notably, *VEGFA* and *PLA2R1*, podocyte-enriched genes (45), showed strong inverse correlations with glomerular injury, highlighting podocyte damage in AAV (**Figure 1F**). In IM/Fib populations, immunoglobulin genes and antigen-presenting molecules such as *HLA-DPB1*, as well as classical complement components (*C1QA*, *C1R*, *C1S*), were upregulated (**Figure 1E**). ECM-related genes (*LUM*, *DCN*, *SERPINF1*, *ADH1B*) were also elevated, with *ADH1B* and *LUM* showing the strongest associations (*R*² > 0.97) with fibrosis and focal glomerulosclerosis, respectively (**Figures 1E, F**). Markers in Myo and vEC/VSMC populations, including *VCAN*, *MGP*, *COL4A1*, *TPM1* and *C3*; *NNMT* and *SOD2*; as well as *SPARCL1* and *TAGLN*, were significantly upregulated in non-focal AAV and positively correlated with fibrotic and glomerular injury indices (**Figure 1E**). *C3* had the highest correlation with fibrosis, while *MGP*, *NNMT*, and TPM1 were linked with glomerular injury (**Figure 1F**).

These cell types and marker associations with renal injury were independently confirmed in a separate adult AAV spatial transcriptomics dataset (**Supplementary Figure S5D-F**), suggesting their potential diagnostic relevance in renal biopsy assessment of AAV (11). ADH1B was not detected in the adult cohort. Neither MGP nor PLA2R1 showed significant differences between disease and control in the adult samples, suggesting potential differences in AAV injury-associated transcriptional features between pediatric and adult patients. Moreover, representative cell-type markers showed broadly consistent expression patterns in both MPO-ANCA and PR3-ANCA subgroups (**Supplementary Figure S7**). Owing to the very limited patient numbers (MPO-ANCA, *n* = 3; PR3-ANCA, *n* = 2), these subgroup analyses are intended as exploratory observations rather than definitive comparisons.

### 3.4 Immune activation and extracellular matrix remodeling in AAV-associated regions

**Figure 3:**
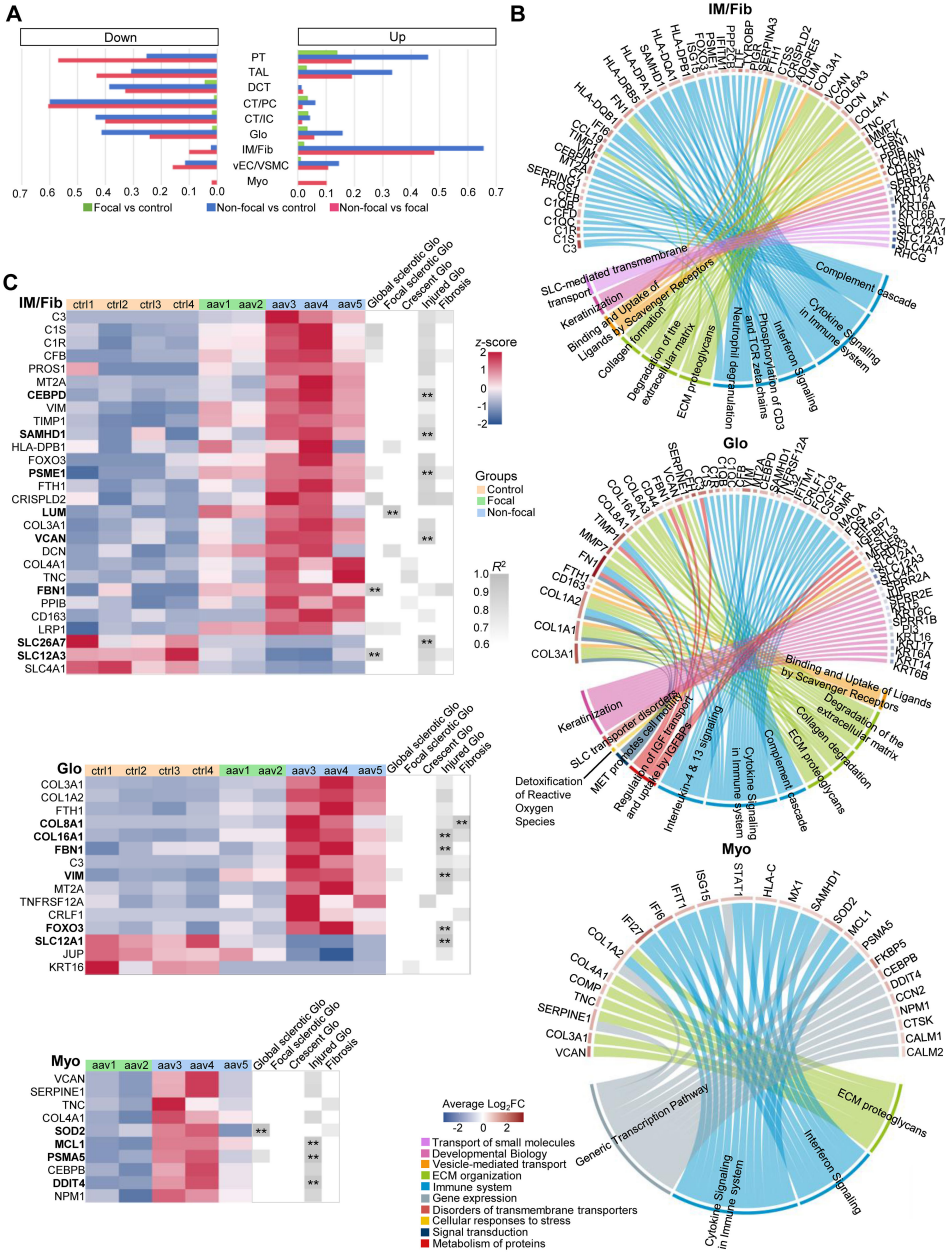
Functions of DEGs in disease-associated regions and their relationships with clinical indicators. **(A)** The bar plot shows the ratios of up/down-regulated DEGs to cell type markers among control, focal, and non-focal groups. **(B)** Circular plots showing the top DEGs in AAV-associated regions (IM/Fib, Myo and Glo) and their associated key biological functions, as identified through Gene Set Enrichment Analysis (GSEA) in the Reactome database. All functions are color-coded on the outer ring to distinguish higher-level Reactome pathway categories, and the average log2 fold change (FC) for each gene is also annotated. **(C)** Heatmap (left) displaying the *z*-score of the expression levels of the top DEGs from AAV-associated regions (IM/Fib, Myo and Glo) in each patient, which are correlated with clinical indicators (*R*² > 0.2, *P*-value < 0.05). The right heatmap illustrates the *R*² values representing the correlation between the DEGs and these clinical indicators. **P-value < 0.01. The DEGs with the most significant correlation with indicators are highlighted in bold font.

We performed pairwise differential expression analysis across control, focal, and non-focal AAV groups in ten different kidney cell types. Disease-associated transcriptional changes were quantified by overlapping DEGs with cell-type-specific markers (**Supplementary Table S3**; **Figure 3A**). AAV-associated regions showed a progressive increase in upregulated marker genes with increasing glomerular involvement, particularly in the IM/Fib compartment (non-focal vs control: 65%; non-focal vs focal: 48%). While most Glo markers were downregulated, a small subset was upregulated in non-focal AAV. Tubular markers were broadly downregulated, except for modest increases in PT and TAL-specific genes.

To explore functional implications of these transcriptional changes, we performed Reactome-based GSEA on the DEGs from AAV-associated regions (non-focal vs control; **Figure 3B**; **Supplementary Figure S8A**) (46). We identified immune activation and ECM remodeling as the most significantly enriched pathways across all compartments. Core immune pathways, including complement activation, cytokine signaling, and interferon responses, were upregulated across the IM/Fib, Glo, Myo, and vEC/VSMC regions. However, the specific DEGs in these enriched pathways varied by cell type. Specifically, the IM/Fib region showed signatures of T cell activation and neutrophil degranulation, consistent with known immune infiltration in AAV (6, 8). In the Glo region, IL-4 and IL-13 signaling was enriched, suggesting a STAT6-mediated immune regulation and renal fibrosis, as previously reported (47).

ECM remodeling pathways, including collagen synthesis, proteoglycan deposition, and matrix degradation, were also broadly upregulated in all AAV-associated regions (**Figure 3B**; **Supplementary Figure S8A**). A conserved set of ECM-related genes (*TIMP1*, *FN1*, *COL3A1*, *VCAN*, *COL6A3*, *COL4A1*, *TNC*, *MMP7*, *FBN1*, *COL1A2*, and *SERPINE1*) was identified across compartments, while others were region-specific (e.g., *LUM*, *DCN* in IM/Fib; *COL8A1*, *CD44* in Glo). Furthermore, the downregulated DEGs were primarily involved in development, transporter function, and protein metabolism, indicating impaired renal function. Correlating DEGs with pathology revealed that genes most strongly associated with clinical indicators (e.g., glomerular injury, fibrosis) were concentrated in IM/Fib, followed by vEC/VSMC, Glo, and Myo (**Figure 3C**; **Supplementary Figure S8B**). Notably, *LUM* (IM/Fib) correlated with focal glomerulosclerosis, while *FBN1* and *SLC12A3* (IM/Fib), *SOD2* (Myo), *C1R* (vEC/VSMC), and *COL8A1* (Glo) were most associated with global sclerosis and fibrosis.

Together, these findings suggest that immune activation and ECM remodeling are central to AAV pathogenesis. Complement and cytokine signaling were widely activated across kidney compartments, along with vesicle-mediated transport. Compartment-specific programs were also observed, including neutrophil degranulation in IM/Fib regions, IL-4/IL-13 signaling in glomerular regions, interferon signaling in Myo regions, and T-cell receptor signaling together with regulation of IGF transport in vEC/VSMC regions.

### 3.5 Shared and distinct renal cell responses in pediatric and adult AAV

**Figure 4:**
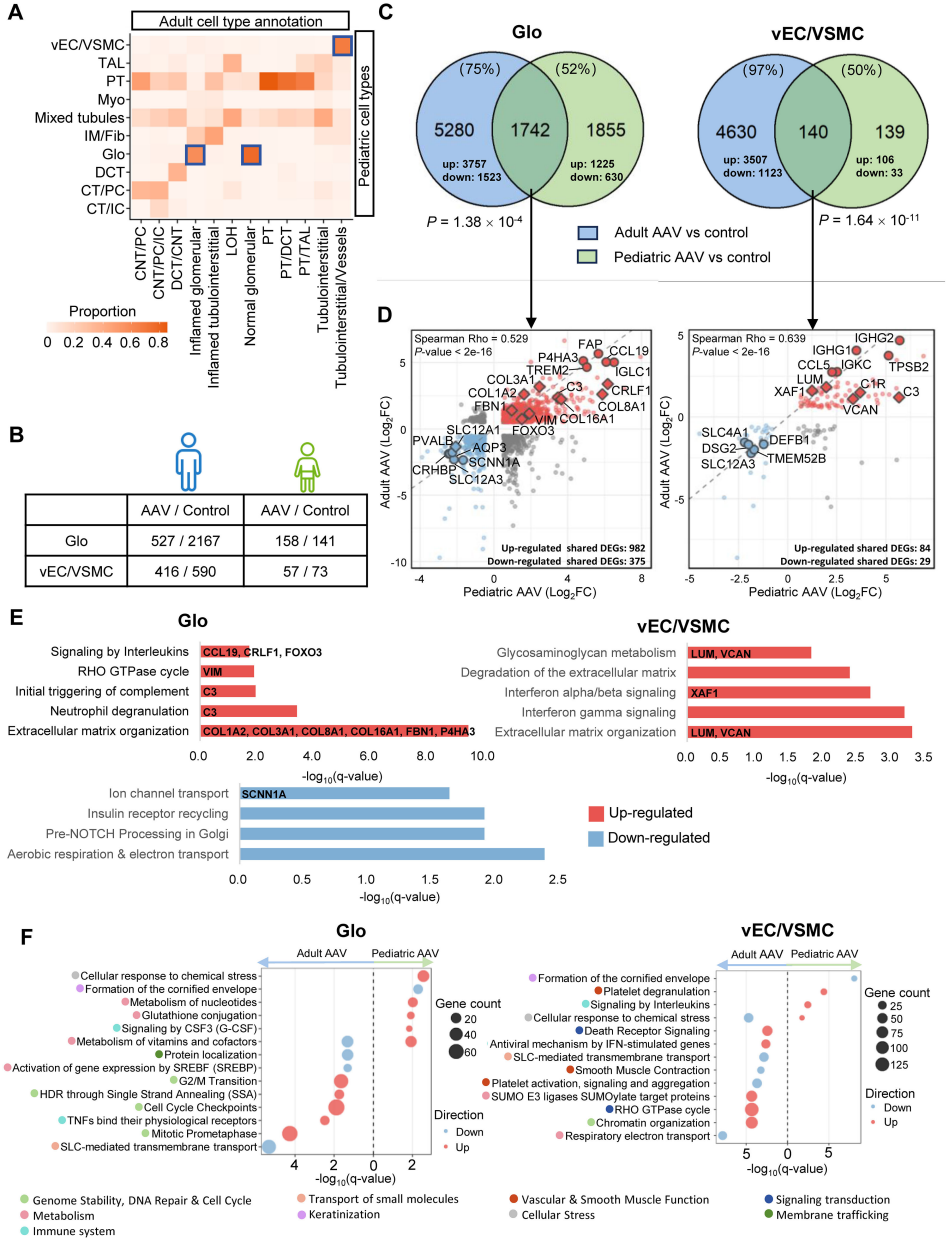
Comparing shared and distinct Glo and vEC/VSMC features between pediatric and adult AAV cohorts. **(A)** Proportional distribution of predicted pediatric AAV cell types across adult annotations. Blue squares indicate renal cell populations consistently identified as AAV-associated in both cohorts. **(B)** Spot counts for Glo and vEC/VSMC in adult and pediatric datasets for AAV versus control. Adult inflamed and normal glomerular populations were consolidated to match the single pediatric Glo annotation. **(C)** Venn diagram of cohort-specific and shared DEGs. Blue: DEGs in the adult cohort; Green: DEGs in the pediatric cohort. The statistical significance of the overlap was calculated by hypergeometric test (*N* = 15342). **(D)** Spearman correlation of shared DEGs (x-axis: pediatric AAV (*n* = 5); y-axis: adult AAV (*n* = 19). Red: upregulated; Blue: downregulated. Solid circles: top five DEGs by correlation (*ρ*) and fold change; diamonds: genes associated with pediatric AAV clinical indicators (**Figure 3C**; **Supplementary Figure S8B**). Dashed line: identity (y = x). **(E)** Bar plot of top five Reactome pathways enriched among shared DEGs, highlighting genes from panel **(D)**. **(F)** Bubble plot of top enriched Reactome pathways from cohort-specific DEGs. Red: upregulated; Blue: downregulated. A central dashed line separates adult-specific (left) and pediatric-specific (right) pathways. All pathways are shown when fewer than five meet significance. The colored solid circle preceding each pathway indicates its corresponding parent category in Reactome dataset.

To determine whether injury-associated programs identified in pediatric AAV are observed in adults, we compared the pediatric dataset with an independent adult spatial transcriptomics cohort comprising 8 controls and 19 AAV patients (**Figure 4**). Cell-type annotations from the pediatric dataset were mapped onto the adult dataset, showing that pediatric IM/Fib compartment corresponded to multiple adult renal compartments, including inflamed glomerular and interstitial regions. However, due to the sparse nature of the pediatric spatial transcriptomics data, further subdivision of IM/Fib resulted in low spot numbers per subgroup and limited statistical power for robust cross-cohort comparisons. Therefore, downstream comparative analyses were restricted to Glo and vEC/VSMC, which exhibited the most stable one-to-one correspondence between the pediatric and adult datasets (**Figures 4A, B**). Differential expression analyses independently identified AAV-associated gene signatures within pediatric and adult cohorts, respectively. Comparison of within-cohort DEG sets revealed significant overlaps between pediatric and adult AAV-associated signatures in both Glo (*P* = 1.38 × 10^-4^, hypergeometric test) and vEC/VSMC (*P* = 1.64 × 10^-^¹¹), indicating shared transcriptional responses to AAV across age groups (**Figure 4C**). Numerous DEGs in Glo and vEC/VSMC associated with pediatric AAV clinical indicators were also differentially expressed in adult AAV samples, suggesting partial cross-cohort reproducibility of disease-associated signals (**Figure 4D**). In Glo, shared upregulated genes mapped to pathways reflecting conserved injury responses, including ECM organization, complement activation, and interleukin signaling (**Figures 3B**, **4E**). The most concordant DEGs—defined by both the highest Spearman correlations in log2FC (AAV/control) and the strongest differential expression—were enriched in ECM remodeling (P4HA3, FAP) and interleukin signaling (CCL19), while other overlapping genes primarily mapped to immune response pathway (TREM2, IGLC1). Shared downregulated genes in Glo included renal transporters (SCNN1A, SLC12A3, SLC12A1, AQP3) (48), neuroendocrine regulator (CRHBP) (49), and calcium-handling marker (PVALB; **Figure 4E**) (50). In vEC/VSMC, shared upregulated pathways involved ECM remodeling and interferon signaling (**Figure 4E**; **Supplementary Figure S8A**), with top DEGs representing conserved inflammatory signatures linked to B cells (IGKC, IGHG1, IGHG2), leukocyte recruitment (CCL5), and mast cell activation (TPSB2) (51). Although no shared downregulated pathways were enriched in vEC/VSMC, several transport (SLC4A1, SLC12A3), structure (DSG2, TMEM52B) (52, 53), and defense-related genes (DEFB1) were consistently decreased in AAV across cohorts (**Figure 4D**).

Pathway analysis of cohort-specific DEGs revealed distinct biological programs between pediatric and adult AAV. Immune-related pathways were upregulated in Glo and vEC/VSMC in both cohorts, but children primarily exhibited neutrophil activation by CSF3 signaling and interleukin-mediated signaling, whereas adults showed TNF-mediated inflammation and IFN-stimulated antiviral response (**Figure 4F**). Moreover, metabolic and cellular stress pathways were enriched in pediatric AAV, whereas they showed reduced enrichment patterns in adult AAV. In contrast, adult AAV showed increased enrichment of genome stability, DNA repair, and cell-cycle pathways in both cell compartments, alongside enhanced death-receptor and Rho-GTPase signaling in vEC/VSMC. Adult AAV patients also displayed downregulation of membrane trafficking, transport, and vascular/smooth-muscle pathways, whereas children uniquely exhibited downregulation of keratinization.

In summary, integrating pediatric and adult kidney spatial transcriptomes revealed a conserved core AAV injury-associated program in glomerular and vascular/smooth-muscle compartments, dominated by ECM remodeling and immune activation with loss of transport and structural genes. However, pediatric AAV showed preferential enrichment of CSF3-driven neutrophil and interleukin signaling with upregulated metabolic and cellular stress pathways, whereas adult AAV showed enrichment of TNF/IFN-centered inflammation, enhanced DNA repair and cell-cycle activity, and suppression of membrane trafficking and vascular programs.

Together, these findings suggest that while the spatial pattern of renal injury is shared across ages, the underlying immunometabolic and stress-response programs exhibit age-related differences.

### 3.6 Network analysis reveals coordinated immune–fibrotic signaling in AAV-associated compartments

**Figure 5:**
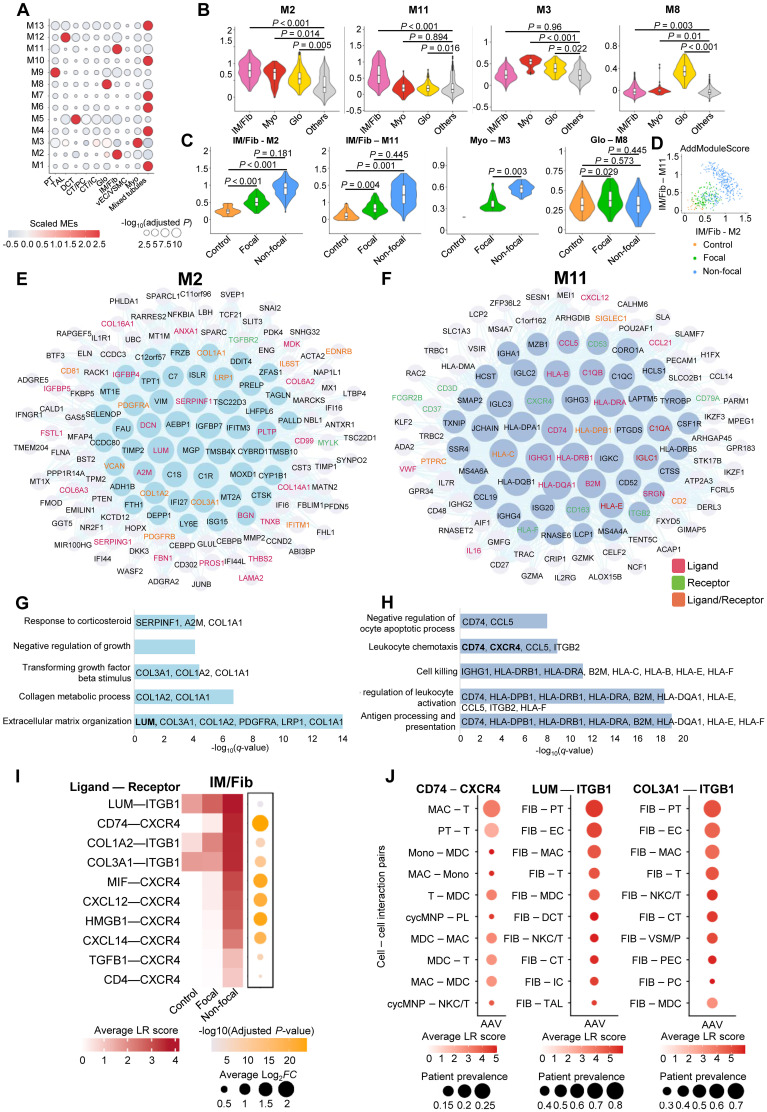
High-dimensional weighted gene co-expression network analysis (hdWGCNA) further characterizes gene co-expression networks in AAV-associated regions from spatial transcriptomic data. **(A)** Thirteen co-expression modules were identified by hdWGCNA in the non-focal AAV group. Correlations between module eigengenes (MEs) and cell type annotations were assessed using Pearson (point-biserial) correlation with BH-adjusted *P*-values. **(B)** Violin plots show expression of module features (top 100 genes) in AAV-associated cell types of AAV cohorts, with M2 and M11 predominantly localized in the IM/Fib region, M3 in the Myo region, and M8 in the Glo region. Feature expression levels were computed using AddModuleScore. *P*-values were determined using Wilcoxon rank-sum test at the sample level. **(C)** Violin plots comparing module feature expression across control, focal, and non-focal groups within each corresponding region. *P*-values were determined using Wilcoxon rank-sum test at the sample level. **(D)** Group-labeled scatter plot showing the correlation between M2 and M11 AddModuleScores in the IM/Fib region. **(E, F)** Network visualizations of eigengenes in M2 and M11. The 50 hub genes are shown as colored circles, with ligands and receptors highlighted. **(G, H)** Top five enriched gene ontology biological process pathways from M2 and M11. Hub gene–derived ligands or receptors involved in each pathway are labeled. **(I)** Heatmap displaying the expression levels of differentially expressed ligand-receptor pairs in the non-focal AAV group, as computed by NICHES. Dot plot illustrating the log2FC and adjusted *P*-values derived from the comparison between AAV (*n* = 3) and the control group (*n* = 4). **(J)** Dot plot showing the top 10 cell–cell ligand–receptor interactions involving CD74–CXCR4, LUM–ITGB1, and COL3A1–ITGB1 in the periglomerular region of adult AAV kidneys (29 AAV and 6 control samples). Only Interactions detected in ≥ 3 patients within each cell-cell pair were retained and ranked by a composite score (see Methods). cycMNP, cycling Mononuclear Phagocyte; PL, plasma cell; pDC, plasmacytoid dendritic cell; MDC, monocyte-derived cell; Mono, monocyte; MAC, macrophage; VSM/P, vascular smooth muscle cells or pericytes; EC, endothelial cell.

To identify coordinated transcriptional programs in AAV-associated regions, we used hdWGCNA on our spatial transcriptomic data from non-focal AAV samples, which yielded 13 distinct gene co-expression modules (**Supplementary Figures S9A, B**). Four of these were enriched in AAV-associated cell types: M2 and M11 in IM/Fib, M3 in Myo, and M8 in Glo (**Figure 5A**). Among these, M11 was highly specific to IM/Fib, while M2 was also moderately active in Myo and Glo (**Figure 5B**). M3 was mainly expressed in Myo, and M8 showed exclusive upregulation in Glo.

We then assessed module activity using eigengenes and AddModuleScores derived from the top 100 hub-ranked genes (kME), and used this metric to compare module activity between focal and non-focal AAV groups. Notably, M2, M3, and M11 activities were higher in the non-focal group, characterized by greater glomerular involvement, across their respective cell types, whereas M8 activity in Glo showed no such trend (**Figures 5C, D**). In all IM/Fib spots, M2 features were higher in the more glomerular-involved group, while M11 features were induced in disease states (**Figure 5D**).

Pathway enrichment and hub gene analysis highlighted M2 and M11 as key networks in IM/Fib (**Figures 5E, F**). Compared to M3 and M8 (**Supplementary Figures S9E–H**), M2 was enriched for ECM organization, featuring hub genes such as *COL1A1*, *COL1A2*, *COL3A1*, *LUM*, *PDGFRA*, and *LRP1* (**Figure 5G**). These genes are involved in collagen biosynthesis and TGF-β signaling, both of which are known drivers of fibrosis in AAV nephropathy (54, 55). The identified response to the corticosteroid pathway may be associated with corticosteroid treatment (13). M11 was related to immune activation, including antigen presentation (*CD74*), leukocyte chemotaxis (*CXCR4*), cytotoxicity, and apoptosis (**Figure 5H**). Cellular communication analysis suggests that *CD74* and *CXCR4* might cooperate in directing immune cell infiltration (**Figure 5I**; **Supplementary Figures S10A, B**). The CD74–CXCR4 interaction being the most significantly enriched among all CXCR4-associated ligand–receptor (L–R) pairs in AAV compared with controls (**Figure 5I**). In IM/Fib, LUM–ITGB1 was the only LUM-associated L–R pair differentially enriched in AAV compared with controls. In addition, the M2 genes COL1A2 and COL3A1 also interacted with ITGB1 through L–R signaling in IM/Fib (**Figure 5I**; **Supplementary Figure S10C**).

To assess whether the ligand–receptor interactions identified in the pediatric IM/Fib compartment were reproducible in an independent cohort, the key L–R genes and AAV injury-related markers showed significant enrichment in the periglomerular regions of the German adult AAV cohort consisting of 6 controls and 32 AAV patients (**Supplementary Figures S11B, C**). Consistent with the predicted CD74–CXCR4 signaling axis, CXCR4 and CD74 were predominantly expressed in immune cells within these periglomerular regions (**Supplementary Figure S11D**).

These key molecules showed positive correlations also in the adult cohort (**Supplementary Figure S11E**). For the CXCR4–CD74 interaction in the periglomerular region, MAC–T cell interaction was the top-ranked cell–cell pair, consistent with the pediatric AAV results, suggesting that CD74–CXCR4 is involved in antigen presentation and leukocyte migration (**Figure 5J**). Similarly, LUM and COL3A1 signaling originated mainly from fibroblasts and targeted proximal tubules, epithelial cells, and macrophages, consistent with the pediatric findings and supporting their association with fibrosis (**Figure 5J**). In addition, the CD74, LUM, and COL3A1 expression were significantly higher in the adult AAV periglomerular region compared with controls, consistent with the pediatric dataset, whereas CXCR4 did not show a significant difference (**Supplementary Figure S11F**). In summary, although the spatial resolution did not allow for the complete separation of immune and fibrotic cells, M2 and M11 showed overlapping spatial features (**Figure 5D**; **Supplementary Figures S9C, D**), suggesting that immune cell infiltration and fibrotic remodeling occur in adjacent or co-localized regions within AAV kidneys.

### 3.7 CXCR4–CD74 crosstalk in IM/Fib mediates leukocyte chemotaxis in AAV

**Figure 6:**
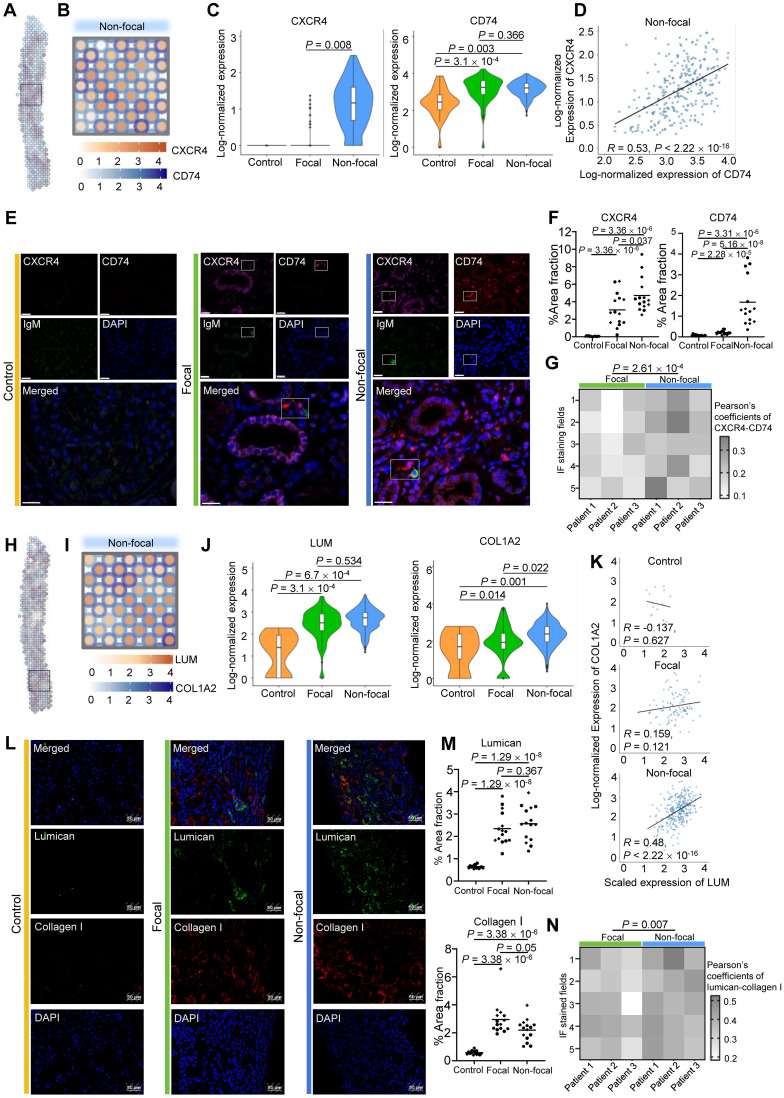
Ligand–receptor interactions reveal leukocyte chemotaxis and ECM remodeling in IM/Fib regions. **(A)** Spatially overlaid and scaled expression of CXCR4 (inner solid circle) and CD74 (outer hollow circle) in a non-focal AAV sample. **(B)** Magnified view of **(A)** showing CXCR4 and CD74 co-expression. **(C)** Violin plots comparing CXCR4 and CD74 expression in IM/Fib across control, focal and non-focal AAV samples (*P*-values by Wilcoxon rank-sum test). **(D)** Scatter plot showing the correlation between CD74 and CXCR4 in co-expressing IM/Fib spots from non-focal AAV, excluding spots with zero expression of either gene. Correlation analysis was not performed for focal AAV due to <10 co-expressing spots. **(E)** Immunofluorescence (IF) staining of CXCR4 (magenta), CD74 (red), and IgM (green) in control, focal, and non-focal samples. White boxes indicate IgM^+^ cells (green) adjacent to CXCR4–CD74 co-localized cells (magenta/red) in representative focal and non-focal AAV patient samples. Scale bars, 20 μm. **(F)** Quantification of CXCR4 and CD74 protein expression (% area fraction) across groups; individual patients (*n* = 3 per group) are denoted by distinct symbols (*P*-values by Wilcoxon rank-sum test). **(G)** Heatmap of Pearson’s co-localization coefficients for CXCR4 and CD74 protein expression in focal and non-focal AAV groups. *P*-values (Wilcoxon rank-sum test) between groups are shown above the heatmap; five IF fields per patient (*n* = 3) are shown. **(H-K)** As in **(A–D)**, measured in LUM and COL1A2. **(L)** IF staining of lumican (green) and collagen I (red) across groups. Scale bars, 50 μm. **(M, N)** Same as in **(F, G)**, measured in lumican and collagen I.

Integration of ligand–receptor and hdWGCNA analyses identified *CXCR4* and *CD74* as hub genes in module M11, associated with immune activation in the IM/Fib region (**Figure 5F**). CD74 and CXCR4 form a receptor complex, and using NICHES for intercellular interaction analyses, we found that cell–cell communication in IM/Fib increased in non-focal compared to focal AAV group (**Supplementary Figure S10A**), with the *CD74*–*CXCR4* axis showing one of the most pronounced activations in non-focal AAV (**Figure 5I**), and enriched for leukocyte chemotaxis, the top pathway in M11 (**Figure 5H**; **Supplementary Figure S10B**). While *CD74* was consistently upregulated across all AAV groups, *CXCR4* was selectively elevated in non-focal AAV (**Figure 6C**). Spatial transcriptomics also revealed the co-expression or adjacency of *CXCR4* and *CD74* in AAV kidneys, particularly in non-focal class (**Figures 6A, B**; **Supplementary Figures S12A, B**). In non-focal AAV, the co-expression of *CXCR4* and *CD74* was strongly positively correlated (**Figure 6D**), suggesting induction of this signaling axis in AAV with greater glomerular involvement, which may mediate leukocyte chemotaxis and subsequent immune activation.

IF analysis corroborated these transcriptomic findings. CD74 and CXCR4 protein expression progressively increased across control, focal, and non-focal AAV, and co-localized near IgM-positive immune cells (**Figure 6E**). *IGHM*, a marker of plasma/memory B cells exclusively expressed in AAV patients, was increased and positively correlated with *CXCR4* in non-focal AAV (**Supplementary Figures S12C–F**). Spatial proximity analysis revealed that CD74/CXCR4 cells were 10–20.5 μm from IgM+ cells in the renal tubulointerstitium (**Supplementary Figures S12G, H**), suggesting that CD74/CXCR4 may signal locally to attract nearby immune cells (a process consistent with paracrine chemotactic recruitment). We also observed higher CXCR4 protein expression in non-focal group compared to focal AAV group, and to a lesser extent for CD74 (**Figure 6F**). The co-localization of CXCR4 and CD74, assessed by Pearson’s coefficients, was significantly higher in non-focal AAV (*P* = 2.61 × 10^-4^; **Figure 6G**), suggesting a stronger interaction in samples with greater glomerular involvement.

In summary, spatial transcriptomic and IF analyses suggest that CXCR4–CD74 co-expression in IM/Fib regions is spatially linked to immune infiltrates and correlates with greater glomerular involvement, supporting their role in leukocyte chemotaxis and immune activation in AAV.

### 3.8 LUM–collagen I/III co-localization correlates with AAV-associated renal fibrosis

In gene co-expression module M2, we identified *LUM* (lumican) as a central hub gene, with pathway and ligand-receptor analyses suggesting its interaction with *COL1A2* and *COL3A1* via *ITGB1*, implicating a role in ECM remodeling (**Figures 5E, G, I**; **Supplementary Figure S10C**). Spatial transcriptomics revealed strong spatial co-expression between LUM and both COL1A2 and COL3A1 within the IM/Fib region, either in the same or adjacent tissue spots (**Figures 6H, I**; **Supplementary Figures S12I-L**). Expression levels of *LUM*, *COL1A2*, and *COL3A1* were associated with the extent of glomerular involvement (**Figure 6J**; **Supplementary Figure S12M**), and all genes were significantly upregulated in AAV compared to controls (**Figure 6K**; **Supplementary Figure S12N**). These data suggest that LUM may contribute to pathological collagen deposition in AAV.

IF staining confirmed increased expression and co-localization of LUM with collagen I and III in AAV kidneys. Lumican and collagen I protein levels were elevated in both focal and non-focal AAV, with significantly stronger co-localization in non-focal cases (*P* = 0.007; **Figures 6L-N**). Similar patterns were observed for collagen III, which also showed enhanced co-localization with LUM in non-focal AAV (*P* = 1.14 × 10^-4^; **Supplementary Figures S12O–Q**). Together, these findings suggest that LUM is associated with fibrotic progression in AAV, potentially through spatial and functional interactions with fibrillar collagens that contribute to ECM accumulation.

## 4 Discussion

Prior AAV studies have largely profiled circulating and infiltrating immune cells, with less attention given to how disease programs are organized within the renal microenvironment. Extensive immune infiltration in glomeruli has been reported (56). Further integration of spatial transcriptomic and scRNA-seq datasets has characterized inflammatory niches and highlighted pathogenic T cells in the kidneys of patients with ANCA-associated glomerulonephritis (11). Building on these studies that identified inflammatory niches, we provide a pilot spatial map of pediatric AAV kidneys that localizes immune–fibrotic activity *in situ*, and links it to histopathological severity at the patient level.

We defined four AAV-associated renal regions, IM/Fib, Glo, Myo, and vEC/VSMC, and identified compartment-specific molecular signatures associated with the extent of glomerular involvement at the patient level. Among these, IM/Fib showed the most pronounced transcriptomic changes, including the highest number of DEGs, cell type markers, and strongest correlations with pathological indicators. The latter were evaluated at the patient level (to avoid pseudoreplication), i.e., for each cell type/region, we generated pseudo-bulk expression per patient and related these to histopathological indices using Spearman rank correlations with FDR control. Complement pathway activation was evident, with increased expression of classical pathway components *C1S* and *C1R* in IM/Fib and *C3* in Myo. While prior work emphasizes alternative-pathway activation in AAV (12), the elevated classical components were observed within IM/Fib are consistent with complement crosstalk in immune-complex-rich niches, potentially contributing to glomerular injury and thromboembolic risk in AAV (57, 58). We also identified *ADH1B* as a potential fibrosis-associated marker in IM/Fib, with minimal expression in controls and a strong patient-level correlation with fibrosis scores. Given ADH1B’s role in aldehyde/retinoid metabolism, we speculate that altered aldehyde handling could contribute to carbonyl stress, fibroblast activation and ECM deposition in AAV (59, 60). Because most representative cell type markers showed differential expression between focal and non-focal AAV in several MPO-ANCA and PR3-ANCA patients in our dataset, these markers may have potential diagnostic value for distinguishing focal from sclerotic/crescentic glomerular lesions within both MPO-ANCA and PR3-ANCA subtypes. This observation remains hypothesis-generating and warrants validation at the protein/activity level and dedicated functional analyses.

Building on these findings, DEGs reflect the direct cellular responses under disease states, highlighting key genes that are markedly up- or downregulated. In contrast, co-expression modules identified by hdWGCNA reveal the coordinated regulatory networks or functional pathways underlying these genes. The immune response-associated module (M11) also includes immunoglobulins, HLA family members, and complement components that are enriched among cell type-specific DEGs in the IM/Fib region of the non-focal AAV group. In M11, the most prominent pathways play a central role in the development of kidney inflammation and renal injury in AAV. During leukocyte activation, ANCA binds to PR3 and MPO, leading to excessive neutrophil activation. These cells release large amounts of pro-inflammatory cytokines, damaging the vascular endothelium (7). Activated monocytes and dendritic cells present antigens via MHC II (*CD74*, *HLA-DBP1*, *HLA-DRB1*, *HLA-DRA*, *HLA-DQA1*), activating T cells and establishing a persistent autoimmune response (6). Vascular endothelial cells and immune cells also secrete chemokines (*CCL19*, *CCL5*, *CCL21*), attracting T cells and dendritic cells to accumulate at sites of inflammation (61, 62). These processes collectively sustain tissue injury and loss of renal function. In addition, negative regulation of neutrophil and lymphocyte apoptosis may lead to sustained tissue damage (**Supplementary Table S7**).

CXCR4 and CD74 were co-expressed and co-localized within IM/Fib neighborhoods. CXCR4 is a vital chemokine receptor implicated in leukocyte trafficking and fibrosis (63, 64). While prior studies support its involvement in kidney fibrosis, the potential interaction between CXCR4 and CD74 in AAV has not been reported. Rather than a classical ligand–receptor pair, CD74 and CXCR4 form a receptor complex that can be engaged by macrophage migration inhibitory factor (MIF) and tuned by CXCL12, promoting chemotaxis and inflammatory signaling (65, 66). Although MIF did not increase uniformly in our dataset, the spatial adjacency of CXCR4/CD74 with IgM^+^ cells and the increased co-localization observed in samples with greater glomerular involvement are consistent with chemotactic recruitment. These features position the CXCR4–CD74 complex as a candidate tissue biomarker and a putative therapeutic axis in AAV, pending functional validation. We will quantify MIF/CXCL12 protein and test perturbations (e.g., blocking antibodies) to establish causality. However, further investigation is needed to identify the immune cell types involved and clarify whether these pathways also contribute to fibrogenesis.

In the ECM remodeling module (M2), key pathways including ECM organization, collagen metabolic processes, and TGF-β signaling were identified as drivers of fibrotic tissue injury (54, 55, 67), which is consistent with findings from DEGs in the IM/Fib region of the non-focal AAV group. Beyond these major fibrosis-related pathways, we also identified pathways associated with the humoral immune response mediated by circulating immunoglobulin (*C1R*, *C1S*, *C7*, *SERPING1*, *CD81*, *SVEP1*) and the response to viruses (*IFITM3*, *IFI27*, *TPT1*, *ISG15*, *DDIT4*) within the M2 network (**Supplementary Table S8**). Interferon-stimulated genes (ISGs) were highly expressed in AAV, indicating abnormal activation of innate immunity and the maintenance of a pro-inflammatory and pro-fibrotic environment (68, 69). Hence, these findings suggest a crosstalk between fibrosis, innate immunity, and chronic inflammation within the AAV-associated region.

A second key finding was the identification of LUM (lumican), a small leucine-rich proteoglycan and structural ECM component, as the hub gene in an ECM remodeling module (M2). *LUM* was strongly co-expressed with collagen genes *COL1A2* and *COL3A1* and spatially co-localized in fibrotic IM/Fib regions. These collagens are early markers of fibrosis (70), and integrin-mediated interactions between LUM and collagen may contribute to excessive ECM deposition. Immunofluorescence confirmed lumican-collagen I/III co-localization and increased expression in non-focal compared to focal AAV. Interestingly, both lumican and collagen were upregulated even in focal AAV, suggesting early involvement in pathogenesis. Although *LUM* is not specific to AAV (being implicated in other kidney fibrotic diseases) (35, 71, 72), its early and progressive upregulation in AAV suggests its potential as an indicator of incipient fibrosis, especially when used in combination with other molecular features.

By integrating pediatric and adult spatial transcriptomic datasets, we identified shared extracellular matrix remodeling and inflammatory programs, suggesting similarities in pathogenic processes across age groups. While this study primarily focuses on pediatric AAV, these findings support the generalizability of key injury-associated programs to adult disease. Despite this conservation, immune and metabolic responses displayed age-associated heterogeneity, highlighting cohort-specific variations that warrant further investigation. Importantly, differential gene expression analyses were performed independently within each cohort by comparing AAV and control samples, and cross-cohort comparisons were based on shared and distinct disease-associated transcriptional programs rather than absolute numbers of detected genes or DEGs. Differences in transcript detection and spot numbers between the independently generated pediatric and adult datasets, which may reflect differences in tissue sampling, biopsy characteristics, and technical factors, should not be interpreted as direct biological differences between age groups.

Moreover, these cell type-specific markers, including CXCR4, CD74, and lumican, may offer added value in enhancing diagnostic granularity and enabling patient stratification in renal biopsies from AAV patients. Unlike conventional histopathological assessments, these transcriptomic signatures showed correlations with the extent of glomerular involvement and pinpointed specific pathological features, including immune infiltration and fibrosis. For instance, *LUM* and its co-localization with *COL1A2*/*COL3A1* may reflect fibrotic remodeling, while *CXCR4*–*CD74* co-expression could be indicative of localized immune activation. These findings were validated in an independent larger German adult spatial transcriptomics cohort, supporting the reproducibility of our results. A less pronounced CXCR4 difference between control and AAV was observed in the adult cohort compared with the pediatric dataset, which may reflect differences in control tissue composition, as tumor nephrectomy-derived controls may exhibit increased baseline CXCR4 expression driven by the tumor microenvironment (73) While preliminary, these findings suggest that integrating such molecular markers with histopathology may refine staging and guide therapies, pending validation in larger independent cohorts. We postulate that these markers constitute candidate tissue biomarkers with complementary roles: diagnostic (refining lesion classification in biopsies) and prognostic (risk stratification by immune activation and fibrotic burden). As the cost of spatial transcriptomics-based assays continues to decline, we envisage a composite spatial severity score (e.g., IM/Fib M11 and M2 module activity combined with marker gene load) to complement conventional histopathology. In addition to the IM/Fib region, we identified additional disease-associated markers in the Myo, Glo, and vEC/VSMC compartments, many of which correlated with pathological features, expanding the landscape of potential diagnostic markers.

Nevertheless, we acknowledge several limitations in our study. First, the rarity of pediatric AAV and the challenge of obtaining high-quality diagnostic biopsies resulted in a small cohort; accordingly, *CXCR4*, *CD74*, and *LUM* as candidate biomarkers remains preliminary. In addition, the spatial transcriptomic cohort did not include patients with mixed Berden class. Although the mixed class also meets the definition of non-focal disease (< 50% normal glomeruli), our analyses were restricted to the enrolled focal and non-focal cases and therefore should not be extrapolated to mixed-class AAV. Furthermore, subgroup analyses according to ANCA serotype remained limited by the small number of MPO-ANCA (*n* = 3) and PR3-ANCA (*n* = 2) patients. These findings should therefore be considered exploratory and hypothesis-generating, requiring validation in larger multicenter cohorts.

Second, biopsy controls with minimal glomerular abnormalities may not fully represent healthy kidney tissue, as subtle molecular alterations may be present despite unremarkable histology. Nevertheless, electron microscopy demonstrated no electron-dense deposits or other ultrastructural features of immune complex–mediated or AAV-related injury, supporting the absence of active glomerular disease. Although mild podocyte foot process effacement was observed, there was no evidence of overt pathological injury, supporting the suitability of these samples as the best available controls for pediatric AAV spatial transcriptomic analyses.

Third, the spatial resolution of the 10x Visium platform used in the pediatric AAV cohort inherently limits cellular resolution because each spot captures transcripts from multiple neighboring cells. Accordingly, the annotated cell types in this study represent the dominant (spot-enriched) cellular composition within each spot rather than true single-cell identities, and the observed cell type–specific transcriptional programs should therefore be interpreted as spot-enriched molecular signatures. Consistent with this limitation, differences between annotation-derived labels and RCTD-inferred compositions likely reflect partial cell-type mixing within spatial transcriptomic spots and differences in resolution and granularity between deconvolution-based estimates and discrete annotation categories, rather than a strict one-to-one correspondence between the two methods. We attempted to mitigate these issues by conducting patient-level analyses (i.e., pseudo-bulk per cell type/region with Spearman correlations, and implementing Benjamini–Hochberg FDR control), applying reference-guided cell-type enrichment analyses using several datasets (e.g., KPMP, DISCO and KCA) and Giotto hypergeometric test, and confirming key findings by conducting blinded immunofluorescence quantification. Similarly, although parietal epithelial cells (PECs), implicated in renal injury and crescent formation, were considered and cell type–specific markers (DKK3, NCAM1, CLDN1, CFH) were used for identification, we were unable to further resolve specific cell lineages within the glomerular (Glo) and interstitial/fibroblast (IM/Fib) regions, likely due to Visium resolution limits, small pediatric glomerular size (fewer than 10 spots per glomerulus), and low PEC abundance (74, 75). An additional limitation relates to the integration of independently generated pediatric and adult spatial transcriptomic datasets. Differential expression analyses were performed independently within each cohort, and cross-cohort comparisons were based on disease-associated transcriptional programs rather than direct comparisons of raw expression values to reduce cohort-specific technical variation. Nevertheless, differences in spatial transcriptomic platforms, sample processing, sequencing depth, cohort composition, and patient numbers may still contribute to technical bias and influence the observed age-associated differences. Consequently, the shared and distinct molecular programs identified across age groups should be interpreted with appropriate caution and validated in larger pediatric and adult cohorts generated using harmonized experimental protocols.

None of the patients had received treatment at the time of biopsy collection (hence minimizing this confounder). Future studies should expand to larger, stratified cohorts based on serotype and age to replicate and extend these findings. Finally, finer cellular resolution will benefit from next-generation platforms such as Xenium, MERFISH, or seqFISH+ and validation of main findings in larger, treatment-annotated cohorts is warranted (76–78).

In summary, we provide a foundational spatial map of AAV kidneys, which extends the previous immune cell subsets analyses. These findings allowed us to generate testable hypotheses and a framework to refine biopsy classification and can help advance our understanding of AAV nephropathy. Future work integrating higher-resolution spatial transcriptomics with multi-omics approaches could further delineate cell-type-specific responses, enabling precise mapping of immune, fibrotic, and parenchymal cell dynamics in AAV nephropathy.

## Data Availability

The datasets presented in this study can be found in online repositories. The names of the repository/repositories and accession number(s) can be found below: https://www.ncbi.nlm.nih.gov/geo/, GSE302677.
