# Supplementary material for: Spatial transcriptomics reveals injury-responsive compartments and coordinated immune–fibrotic signaling in ANCA-associated renal vasculitis
Source: Front Immunol. 2026 Jul 31;17:1884100. doi: 10.3389/fimmu.2026.1884100 (PMC13473267; doi:10.3389/fimmu.2026.1884100)
Supplement: Supplementary file 1 [file Table1.docx]

***Supplementary Materials***

**This PDF file includes:**

Supplementary Methods

Supplementary Figures (Figs. S1 to S12)

**Supplementary Methods**

**Hypergeometric test-based validation of cell type annotations**

To validate cell type annotations, we performed hypergeometric test-based enrichment analysis using the Giotto package (v3.3.0) [1]. For each group (control, focal and non-focal), Giotto objects were generated with Seurat metadata. References (table S9) were restricted to scRNA-seq data from healthy kidneys and cortex-derived cell types to avoid disease-related bias. Cell-type-specific marker genes were identified using Seurat’s “FindAllMarkers” function (MAST method) from the Kidney Cell Atlas (KCA) mature kidney dataset and healthy kidney data in the Deeply Integrated Single-Cell Omics (DISCO) database (table S9) [2, 3]. Marker genes cutoff: log_2_FC ≥ 0.25 and adjusted *P*-value ≤ 0.05 (Wilcoxon rank sum test). We performed hypergeometric testing using Giotto's "runHyperGeometricEnrich" function to generate enrichment scores, defined as −log_10_(*P*-value) per spatial spot. Mean scores for each annotated cell type were visualized as a heatmap.

**Spatial Deconvolution Analysis**

Spatial deconvolution was performed using Robust Cell Type Decomposition (RCTD) method from the spacexr package (v2.0.0) [4], with healthy kidney tissue from the KCA serving as the scRNA-seq reference (25 annotated cell types, table S9). Because RCTD incorporates spatial coordinates, deconvolution was conducted separately for the control,focal AAV, and non-focal AAV sections. For each section, cell type proportions were obtained from scRNA reference, and Immune cells were defined as the sum of all immune-related cell types. The waffle package (v1.0.2) was used to visualize the top five deconvoluted cell types (by proportion) from the scRNA reference within each annotated spatial transcriptomics cell type. The average proportions across all spots were expressed as percentages, with each square representing 1% of the total composition.

**Evaluation of Deconvolution Accuracy Using Jaccard Index**

To evaluate the accuracy of spatial transcriptomics deconvolution, previously obtained RCTD-derived cell type proportion estimates for each spatial spot were used. Cell type–level proportions were aggregated into predefined categories to match the pediatric annotation schema, including CT/IC (intercalated cell), CT/PC (principal cell), DCT (connecting tubule), Glo (glomerular endothelium and podocyte), IM/Fib (immune cells and fibroblasts), Myo (myofibroblast), PT (proximal tubule), TAL (thick ascending limb of the loop of Henle), and vEC/VSMC (ascending vasa recta endothelium, descending vasa recta endothelium, and peritubular capillary endothelium).For each spot, the cell type with the highest aggregated proportion was assigned as the predicted label. The Jaccard index was used to quantify the overlap between sets of spots assigned to each category under the two annotation strategies, serving as a measure of agreement between predicted labels and pediatric cell type annotations and providing a quantitative assessment of concordance with prior biological knowledge [5, 6]. Epithelial progenitor cells were excluded from the calculation, as these partially undifferentiated cells can appear across multiple cell types and may confound annotation (fig. S3C). Finally, Jaccard indices were summarized for each annotated cell type and visualized as a bar plot to illustrate overall deconvolution performance.

**Human AAV Spatial Transcriptomics Reference Dataset Validating Key Features of AAV-responsive Regions**

A recently available human AAV spatial transcriptomics reference confirmed the characteristics of AAV-responsive regions in our dataset (GSE250138, table S9) [7]. The reference datasets comprised 10 predefined normal renal cell types (including CT/PC, CT/PC/IC, DCT/CT, Glo, LOH, PT, PT/DCT, PT/TAL, tubulointerstitium, and vasculature) and 2 AAV-responsive cell populations (inflamed Glo and tubulointerstitium), derived from 19 ANCA-crescentic glomerulonephritis (GN) patients and 8 controls (table S9). We evaluated the similarity between our spatial transcriptomics data and the reference’s gene expression profiles using the fgsea package (v1.26.0). The DEGs identified by the “FindMarkers” function in the reference dataset (between controls and ANCA-GN patients, with log_2_FC ≥ 1 and adjusted *P*-value ≤ 0.05) and the up-regulated DEGs from our AAV severe group (ranked by log_2_FC) were used as input for analysis. The NES was then calculated to show the enrichment of cell-type features from the reference dataset within our cell types. The key cell type markers identified in our AAV-responsive regions were further validated within the spatial reference dataset.

**Comparative Cross-Dataset Analysis**

Spatial transcriptomics profiles from the pediatric AAV cohort were compared with the independent adult AAV dataset described above (GSE250138, table S9). To ensure consistency for downstream analysis, pediatric cell type annotations were transferred to the adult dataset using the Seurat’s “FindTransferAnchors” and “TransferData” workflows, followed by validation against the adult dataset’s original annotations. Because the original adult annotations did not include fibrosis-related cell types, the comparative analysis were restricted to shared glomerular and vascular compartments present in both cohorts (table S10).

Differential gene expression analysis was performed separately within the adult and pediatric cohorts. In the adult dataset, DEG analysis was performed for Glo and vEC/VSMC cell types by comparing AAV with control samples using Seurat’s “FindMarkers” function (MAST method). An analogous within-cohort analysis was conducted in the pediatric dataset. The resulting DEGs were benchmarked against the pediatric AAV DEGs, using criteria of |log_2_FC| ≥ 0.5 and adjusted *P*-value ≤ 0.05. Overlapping and cohort-specific DEGs were visualized using VennDiagram (v1.7.3), and the statistical significance of overlap was calculated using the hypergeometric test. Shared DEGs were subjected to Spearman correlation analysis to evaluate concordance in expression changes across cohorts, and their log_2_FC values were displayed in Scatter plots. Reactome pathway enrichment for commonly upregulated and downregulated DEGs was summarized using bar plots, whereas cohort-specific signatures were represented using bubble plots. The thresholds are aligned with those applied in the ORA described in the Functional Enrichment Analysis section.

**Supplementary Figures**

Fig. S1. Collagen IV α5 chain immunofluorescence staining (IF) and electron microscopy (EM) findings for all control patients included in this study. Collagen IV α5 chain immunofluorescence staining demonstrated a normal staining pattern. Electron microscopy revealed focal effacement of podocyte foot processes involving approximately 30–40%. No definite electron-dense deposits were identified in any glomerular compartments. Scale bars: 50 μm (IF) and 2 μm (EM).

**Fig. S2. Quality control, annotation, and cell type validation in the integrated data.** (**A**) The QC metrics across groups and patients were shown in the violin plot (Wilcoxon rank-sum test). (**B**) The ratio distribution of sequenced spots from each patient across cell types. (**C**) Giotto hypergeometric tests with two human kidney scRNA-seq reference datasets from the Deeply Integrated human Single-Cell Omics (DISCO) and Kidney Cell Atlas (KCA) databases across all clusters in the integrated datasets. The enrichment score is calculated based on the *P*-value from the hypergeometric test, **–**log_10_(*P*-value), and finally applying *z*-score normalization. LOH, Loop of Henle; PT, proximal tubule; DT, distal tubule; ICA, intercalated cell type A; ICB, intercalated cell type B; EC, endothelial cell; TAL, thick ascending limb; EPC, epithelial progenitor cell; CT, connecting tubule; PC, principal cell; IC, intercalated cell; GE, glomerular endothelium; AVRE; ascending vasa recta endothelium; DVRE, descending vasa recta endothelium; PEC, peritubular capillary endothelium; DC, dendritic cell; pDC, plasmacytoid dendritic cell. (**D**) The QC metrics across cell types from the integrated dataset were shown in the violin plot. (**E**) Dot plot representing gene expression of canonical cell type markers in integrated datasets. (**F**) Heatmap of top 5 gene ontology biological process (GOBP) pathways for each cell type and top shared GOBP pathways among cell types in integrated datasets by GO over-representation analysis.

Fig. S3. Spatial transcriptomic profiling reveals cell type-specific markers distribution in the non-focal AAV group. The top-left panel shows spatial cell type annotations in the non-focal AAV group. All remaining panels display the spatial expression of marker genes for specific cell types, including tubular (*ALDOB, SLC12A1, SLC12A3, AQP2, SLC26A7*), glomerular (*PODXL*), immune (*IGHG1, CD163*), smooth muscle cell (*MYH11*) and fibroblast markers (*COL8A1*). Each marker gene is preceded by a solid dot label, which is color-coded to match the corresponding cell type in the annotation panel. Color intensity reflects scaled gene expression across spatial spots.

Fig. S4. Evaluation of the consistency between RCTD deconvolution based on the scRNA reference and cell types annotated by unsupervised clustering. (A) Bar plot showing the Jaccard index for each annotated cell type, representing the concordance between Visium annotations and RCTD deconvolution-derived spot assignments. Predicted spot labels were obtained by aggregating deconvolution-derived scRNA-seq cell type proportions into predefined Visium cell types and assigning each spot to the cell type with the highest aggregated proportion (detailed in Methods). (B) Waffle plots displaying the top five RCTD-deconvoluted scRNA cell types, ranked by their proportions, within each annotated spatial transcriptomics cell type. Each square corresponds to 1% of the total cell-type proportion. Podocytes and glomerular endothelium are both cell types of the glomerulus. (C) The dot plot presents the Top 50 epithelial progenitor cell signature genes (as defined in the KCA literature) across all annotated cell types. These signature genes are detected in almost all cell types, indicating low cell-type specificity. Hence, epithelial progenitor cells appear in all deconvolution results.

**Fig. S5. Validation of injured cell types in AAV.** (**A**) UMAP showing unsupervised clustering with cell type annotations in split datasets (ctrl, focal, non-focal). PT, proximal tubule; TAL, thick ascending limb; DCT, distal convoluted tubule; CT/PC, connecting tubule/principal cell; CT/IC, connecting tubule/intercalated cell; Glo, glomerulus; IM/Fib, immune cell and fibroblast; vEC/VSMC, vascular endothelial cell/vascular smooth muscle cell; Myo, myofibroblast. (**B**) Dot plot of injury marker expression in AAV- associated regions across patients. (**C**) Heatmap of Spearman correlation coefficients between renal pathological indicators and cell type proportions across five AAV patient samples. * *P*-value ≤ 0.1, ** *P*-value ≤ 0.05. (**D**) Heatmap of GSEA normalized enrichment scores (NES) for representative differentially expressed genes (DEGs) (log_2_FC ≥ 1) across cell types in a spatial transcriptomics reference dataset (control vs. AAV), based on ranked DEGs (log_2_FC ≥ 0.25) from each cell type in our datasets (control vs. non-focal). The x-axis denotes our cell types and the y-axis denotes reference cell types; black boxes indicate gene features of two reference cell types with the highest BH-adjusted *P*-value in our AAV-associated regions. (**E-F**) Violin plots comparing expression of cell type markers (from Fig. 2F) across control, AAV normal, and AAV inflamed tubulointerstitium and glomerulus regions in the reference dataset. *P*-values were determined using Wilcoxon rank-sum test at the sample level. Adjusted *P* values were calculated using the BH method. Statistical significance is indicated as follows: ns, not significant; **P*_adj < 0.05; ***P*_adj < 0.01; ****P*_adj < 0.001; *****P*_adj < 0.0001.

**Fig. S6. Spatial feature plots illustrating the expression patterns of cell type markers from AAV-associated regions.** Representative spatial feature plots showing the expression of marker genes from IM/Fib, Myo, Glo and vEC/VSMC cell types. For each marker gene, the left, middle, and right panels correspond to tissue sections from the control, focal, and non-focal AAV groups, respectively. Blue circles highlight each AAV- associated region with elevated gene expression. Color intensity indicates the scaled gene expression across spatial spots.

**Fig. S7. Violin plots showing representative pathological indicator**–**related cell type markers in AAV-associated regions (see Fig. 2F), comparing focal and non-focal disease in MPO-ANCA and PR3-ANCA patients.** Patient classification details are provided in table S1.

**Fig. S8. Functions of differentially expressed genes (DEGs) in the vEC/VSMC region and their relationships with clinical indicators.** (**A**) Circular plots showing the vEC/VSMC and their associated key biological functions, as identified through Gene Set Enrichment Analysis (GSEA) in the Reactome database. All functions are color-coded on the outer ring to distinguish higher-level Reactome pathway categories, and the average log2 fold change (log2FC) for each gene is also annotated. (**B**) Heatmap (left) displaying the *z*-score of the expression levels of the top DEGs from vEC/VSMC in each patient, which is correlated with clinical indicators (*R*² > 0.2, *P*-value < 0.05). The right heatmap illustrates the *R*² values representing the correlation between the DEGs and these clinical indicators. ** *P*-value ≤ 0.01.

**Fig. S9. The hdWGCNA analysis of the non-focal AAV group showed the key modules in AAV- associated regions.** (**A**) Optimal soft threshold selection. The power value equaled 9 when the network reached a scale-free distribution. (**B**) There are 13 modules identified by the hdWGCNA clustering tree with an optimal soft threshold of 9. (**C**-**D**) Spatial mapping of module activity using SpatialFeaturePlots, showing tissue distributions of M2 and M11 features generated by AddModuleScore. (**E-F**) Network visualizations of eigengenes in M3 and M8. The 50 hub genes are shown as colored circles, with ligands and receptors highlighted. (**G-H**) Top five enriched gene ontology biological process (GOBP) pathway from M3 and M8. Hub gene–derived ligands or receptors involved in each pathway are labeled.

**Fig. S10. Ligand–receptor interactions from NICHES.** (**A**) Networks show overall cellular communication. Nodes indicate different regions of each group, while edges represent the number of interactions within (autocrine interactions) or between (paracrine interactions) regions. Other cell types are non- involved region. (**B**) Over-Representation Analysis (ORA) enrichment of GOBP pathways associated with ligand-receptors in the M11. Hub ligands or receptors from M11 are annotated. (**C**) ORA enrichment of GOBP pathways associated with ligand-receptors in the M2. Hub ligands or receptors from M2 are annotated.

**Fig. S11. Validation of the selected ligand–receptor (L–R) pairs in an external German adult AAV Xenium cohort (control, *n* = 6; AAV, *n* = 32).** (A) Visual representation of the glomerular (Glo) and periglomerular (Periglo) regions delineated in the adult Xenium dataset. All other remaining tissues are classified as the tubulointerstitial (TI) area. (B) Heatmap showing distinct spatial expression patterns of key L–R genes across renal compartments in control and AAV cohorts. (C) Dot plot showing expression of injury-, immune- and fibrosis-related genes across renal compartments in control and AAV cohorts. (D) Dot plot representing expression of key L–R genes across cell types in the periglomerular region of control and AAV cohorts. (E) Scatter plots showing the expression correlations for two gene pairs (CD74/CXCR4 and LUM/COL3A1) within the periglomerular region of the AAV cohort, excluding spots with zero expression. (F) Violin plots comparing expression of key L–R genes in top-ranked cell types within the periglomerular region between control (*n*=6) and AAV (*n*=29) patients. *P*-values were calculated using Wilcoxon rank-sum test. cycNKC/T, cycling NK cytotoxic T cell; cycMNP, cycling Mononuclear Phagocyte; cDC, conventional dendritic cell; PL, plasma cell; pDC, plasmacytoid dendritic cell; MDC, monocyte-derived cell; Mono, monocyte; MAC, macrophage; N, neutrophil; NEU, neuronal cell; PpE, papillary tip epithelial cells abutting the calyx; DTL, descending thin limb of loop of Henle; ATL, ascending thin limb of loop of Henle; VSM/P, vascular smooth muscle cells or pericytes; EC, endothelial cellMC, mesangial cell; POD, podocyte.

**Fig. S12. Expression and spatial distribution of stained ligand-receptor pairs.** (**A**) Spatially overlaid and scaled expression of *CXCR4* (inner solid circle) and *CD74* (outer hollow circle) in a focal AAV sample. (**B**) Magnified view of (**A**) showing *CXCR4* and *CD74* co-expression. (**C**) Violin plots comparing *IGHM* expression in IM/Fib across control, focal, and non-focal AAV samples (*P*-values by Wilcoxon rank-sum test). (**D**) Scatter plots showing the correlation between *CXCR4* and *IGHM* expression in the IM/Fib region of non-focal AAV. (**E**-**F**) As in (**A**-**B**), showing spatial co-expression of *CXCR4* and *IGHM* in a non-focal AAV sample. (**G**-**H**) Immunofluorescence (IF) images showing magnified views from Fig. 5E (merged channels) in focal and non-focal AAV samples. Labels indicate nucleus-to-nucleus distances between IgM-positive cells (green) and CXCR4-CD74 co-localized cells (magenta/red). Scale bars, 5 μm. (**I**-**J**) As in (**A**-**B**), showing spatial co-expression of *LUM* and *COL1A2* in a focal AAV sample. (**K**-**L**) As in (**A**-**B**), showing spatial co-expression of *LUM* and *COL3A1* in a non-focal AAV sample. (**M**) As in (**C**), measured in COL3A1. (**N**) Scatter plots showing correlations between *LUM* (x-axis) and *COL3A1* (y-axis) expression in the IM/Fib region of control, focal, and non-focal AAV groups. (**O**) IF staining of lumican (green) and collagen III (red) in control, focal, and non-focal samples. Scale bars, 50 μm. (**P**) Quantification of lumican and collagen III protein expression (% area fraction) across groups (*P*-values by Wilcoxon rank-sum test). (**Q**) Heatmap of Pearson’s co-localization coefficients for lumican and collagen III protein expression in focal and non-focal AAV groups. *P*-values (Wilcoxon rank-sum test) between groups are shown above the heatmap; five IF fields per patient (*n* = 3) are shown.

**References**

1.	Dries R, Zhu Q, Dong R, Eng C-HL, Li H, Liu K, et al. Giotto: a toolbox for integrative analysis and visualization of spatial expression data. Genome Biol. 2021;22:1–31.

2.	Li M, Zhang X, Ang KS, Ling J, Sethi R, Lee NYS, et al. DISCO: a database of Deeply Integrated human Single-Cell Omics data. Nucleic Acids Res. 2022;50(D1):D596–D602.

3.	Stewart BJ, Ferdinand JR, Young MD, Mitchell TJ, Loudon KW, Riding AM, et al. Spatiotemporal immune zonation of the human kidney. Science. 2019;365(6460):1461–6.

4.	Cable DM, Murray E, Zou LS, Goeva A, Macosko EZ, Chen F, et al. Robust decomposition of cell type mixtures in spatial transcriptomics. Nat Biotechnol. 2022;40(4):517–26.

5.	Jaccard P. Étude comparative de la distribution florale dans une portion des Alpes et des Jura. Bull Soc Vaudoise Sci Nat. 1901;37:547–79.

6.	Tang M, Kaymaz Y, Logeman BL, Eichhorn S, Liang ZS, Dulac C, et al. Evaluating single-cell cluster stability using the Jaccard similarity index. Bioinformatics. 2021;37(15):2212–4.

7.	Engesser J, Khatri R, Schaub DP, Zhao Y, Paust H-J, Sultana Z, et al. Immune profiling-based targeting of pathogenic T cells with ustekinumab in ANCA-associated glomerulonephritis. Nat Commun. 2024;15(1):8220.
